# Hybrid mesh for magnetotelluric forward modeling based on the finite element method

**DOI:** 10.1038/s41598-023-27758-2

**Published:** 2023-01-11

**Authors:** Nian Yu, Xialan Wu, Xinyu Liu, Ruiheng Li, Hongye Zhang, Lei Gao

**Affiliations:** 1 School of Electrical Engineering, Chongqing University, Chongqing, 400044 China; 2 Hubei University of Economics, Wuhan, 430205 Hubei China; 3 State Key Laboratory of Power Transmission Equipment and System Security and New Technology, Chongqing, 400044 China

**Keywords:** Geomagnetism, Geophysics

## Abstract

Unstructured tetrahedral grids have been applied in magnetotelluric (MT) forward modeling using the finite element (FE) method because of their adaptability to complex anomalies. However, high-quality results require an extreme refinement of the near-surface area, which leads to excessive meshes and an increased degree of freedom (DoF) of the governing equation of the finite element system. To reduce the computational cost, we have developed a hybrid mesh based on triangular prisms and tetrahedrons. The required elements in the near-surface area are reduced because the quality of the triangular prism is not limited by the element aspect ratio. The deep area is discretized by tetrahedral elements to ensure the flexibility of the unstructured grids. The superiority of this hybrid mesh has been tested on a layered model, the DTM1 model and terrain relief models. The results show that the modeling efficiency has been improved, especially for high-frequency data. The accuracy of the modeling using the hybrid mesh is significantly higher than that of the tetrahedral mesh with a similar DoF. Usage of the hybrid mesh can be easily adapted to complex geoelectric models with strong terrain fluctuations, which requires less computational cost than using conventional unstructured elements.

## Introduction

Unstructured grids with tetrahedral elements are suitable for dividing complex underground anomalous bodies in terms of topography and bathymetry because they can fit arbitrary shapes of geological bodies well^1–5^. This type of grid has become a useful tool for discretizing the geoelectric model in the numerical simulation of three-dimensional geophysical electromagnetic (EM) fields using the finite element method (FEM)^6–8^ and has been widely used in magnetotelluric (MT) forward modeling^9–11^. However, unstructured grids often need to be refined into extremely small elements to avoid ill-conditioning^12,13^. The refinement often results in too many redundant elements and reduces the computational efficiency of the FEM forward modeling.

To reduce the number of required elements, a scheme of nonconforming grids has been used to discretize the geoelectric model^14,15^. This kind of regular grid, whose element quality is not restricted by the aspect ratio of the element, has a variable scale and stable local refinement capabilities^16,17^. It helps to reduce the degrees of freedom (DoF) and is suitable for large-scale 3-D geophysical EM forward problems, such as MT forward modeling. A multiresolution approach suggested by Cherevatova et al.^13^ allows grid refinement only in the horizontal directions and keeps the degree of refinement in the vertical direction constant. The stretching grid obtained from this multiresolution approach can help to improve calculation accuracy with fewer elements in the near-surface area . In addition, the longer the element is stretched, the more elements are saved, and the decrease in numerical accuracy caused by element stretching can be compensated by a higher-order element processing scheme^18,19^. However, some elements of the stretched grid with hanging nodes cannot share just one whole edge or face^13,14^, and the convenience and flexibility of mesh generation are not as good as traditional tetrahedral elements.

Triangular prism elements can be used to build the stretched grid in the near surface area^20^. However, the accuracy of the numerical solution will be reduced when the stretched grid extension is too long. In this research, second-order shape functions are used to discretize near-surface prismatic elements. The deep region is still freely discretized by the tetrahedral elements. Because triangular prism elements and tetrahedral elements can share a triangular surface, we developed a hybrid mesh with second-order shape functions containing two kinds of elements.

This paper is structured as follows. We first introduce our governing equations for forward modeling, the $\mathbf {A}$-$\varphi$ system with gauge fixing. Then, we describe how to create a hybrid mesh with triangular prism elements and tetrahedral elements and how we apply it to FEM forward modeling. We use a layered model to preliminarily illustrate the advantages of this method in terms of calculation efficiency and accuracy. The advantages of the hybrid mesh are further demonstrated by three types of numerical examples. The conclusion is in the last section.

## Theoretical background

When the displacement current is ignored in the quasistatic approximation and the time dependence is assumed to be $e^{i \omega t}$, the governing equations for the induced EM fields are written as:

1 $$\begin{aligned}&\nabla \times \mathbf{{E}} + i\omega \mu \mathbf{{H}} = 0 \end{aligned}$$

2 $$\begin{aligned}&\quad \nabla \times \mathbf{{H}} - \sigma \mathbf{{E}} = 0 \end{aligned}$$

where **E** and **H** are the induced electric and magnetic fields, respectively. $\omega$ is the angle frequency. $\sigma$ and $\mu$ are the conductivity and permeability of the EM medium, respectively. By substituting Eq. (2) into the formula after taking the curl of Eq. (1), the Helmholtz equation of the electric field **E** can be obtained as:

3 $$\begin{aligned} \nabla \times \nabla \times \mathbf{{E}} + i\omega \mu \sigma \mathbf{{E}} = 0 \end{aligned}$$

### Governing equation

The electric field can be decomposed into the magnetic vector potential **A** and electric scalar potential $\varphi$. This can reduce the weakness of the curl-curl electric equation^21–24^, accelerate the speed of solving the FE system with an iterative solver and obtain higher numerical accuracy. The relation of the potentials and fields can be represented as:

4 $$\begin{aligned} \mathbf{{E}} = - i\omega \mathbf{{A}} - \nabla \varphi \end{aligned}$$

5 $$\begin{aligned} \nabla \times \mathbf{{A}} = \mu \mathbf{{H}} \end{aligned}$$

Substituting Eq. (4) into Eq. (3) results in the following equation^25^:

6 $$\begin{aligned} \nabla \times \nabla \times \mathbf{{A}} + i\omega \mu \sigma \mathbf{{A}} + \mu \sigma \nabla \varphi = 0 \end{aligned}$$

In conjunction with **J** = $\sigma$**E**, by substituting Eq. (4) into the Gaussian divergence equation, the current divergence is zero and can be expressed as follows:

7 $$\begin{aligned} \nabla \cdot \left( { - i\omega \sigma \mathbf{{A}} - \sigma \nabla \varphi } \right) = 0 \end{aligned}$$

When both the curl and divergence of **A** are given, **A** is uniquely defined up to a constant. Therefore, to obtain a unique solution, we add the Coulomb gauge condition^26,27^:

8 $$\begin{aligned} \nabla \cdot \mathbf{{A}} = 0 \end{aligned}$$

The Coulomb gauge is imposed to strengthen the divergence limit and enhance the uniqueness of the FE equations. On the basis of Eq. (6) derived from Ampere’s rule, we add a Lagrange multiplier term to eliminate the external current density divergence^21,23^. Equations (6) and (7) can be rewritten as follows:

9 $$\begin{aligned}&\nabla \times \nabla \times \mathbf{{A}} + i\omega \mu \sigma \mathbf{{A}} + \mu \sigma \nabla \varphi = \nabla \lambda \end{aligned}$$

10 $$\begin{aligned}&\quad \nabla \cdot \left( { - i\omega \sigma \mathbf{{A}}} \right) = 0 \end{aligned}$$

In Eq. (10), −i$\omega \sigma$ is constant, so the divergence of the magnetic vector potential is specified.

For the geophysical EM field propagation problem, we use homogeneous Dirichlet boundary conditions in the truncated model boundary.

### Solving a linear system of equations

We use the Galerkin method to discretize Eq. (9). The discretized expression is as follows:

11 $$\begin{aligned} \begin{array}{l} \int _\Omega {\left( {\nabla \times \mathbf{{N}}} \right) \cdot \left( {\nabla \times {\varvec{{\tilde{A}}}}} \right) } d\Omega - \int _{\gamma + \Gamma } {\mathbf{{N}} \times \left( {\nabla \times {\varvec{{\tilde{A}}}}} \right) } \cdot \hat{n}dS + i\omega \mu \int _\Omega {\sigma \mathbf{{N}} \cdot {\varvec{{\tilde{A}}}}} d\Omega + \\ \mu \int _\Omega {\sigma \mathbf{{N}} \cdot \nabla \tilde{\varphi }} d\Omega = \int _\Omega {\mathbf{{N}} \cdot \nabla \tilde{\lambda }} d\Omega \end{array} \end{aligned}$$

where $\mathbf {N}$ denotes the vector shape function. ${{\varvec{{\tilde{A}}}}}$, ${\tilde{\varphi }}$, and ${\tilde{\lambda }}$ are the approximate solutions of $\mathbf{{A}}$, $\varphi$, and $\lambda$, respectively. $\Omega$ is all domains of the calculation model. $\Gamma$ and $\gamma$ are the outer and inner boundaries of the mesh, respectively. Similarly, the discrete forms of Eqs. (7) and (8) are obtained as follows^23^:

12 $$\begin{aligned} i\omega \int _\Omega {\nabla N \cdot \left( {\sigma {\varvec{{\tilde{A}}}}} \right) } d\Omega + \int _\Omega {\nabla N \cdot \sigma \nabla \tilde{\varphi }d\Omega } - \int _{\gamma + \Gamma } {N\sigma \nabla \tilde{\varphi }\cdot \hat{n}dS} = 0 \end{aligned}$$

13 $$\begin{aligned} - \int _\Omega {\nabla N \cdot {\varvec{{\tilde{A}}}}d\Omega } = 0 \end{aligned}$$

where N denotes the scalar shape function. We use natural electromagnetic field sources to form the right side of the system. When the polarization direction of the magnetic field source is the y direction, the air top interface loads the magnetic field source $H_{0}$ = (0, 1, 0), the boundary condition of the outer boundary parallel to the x-axis is $\hat{n} \times (1/\mu )\nabla \times \mathbf{{A}} = 0$, and the boundary conditions of the outer boundary perpendicular to the x-axis and the bottom boundary are $\hat{n} \times \mathbf{{A}} = 0$. When the polarization direction of the magnetic field source is the x direction, the air top interface loads the magnetic field source $H_{0}$ = (1, 0, 0), the boundary condition of the outer boundary perpendicular to the x-axis is $\hat{n} \times (1/\mu )\nabla \times \mathbf{{A}} = 0$, and the boundary condition of the outer boundary and the bottom boundary parallel to the x-axis are $\hat{n} \times \mathbf{{A}} = 0$. The boundary condition of $\varphi$ is ${\varphi _\Gamma } = 0$. Add the relevant boundary conditions through the second term in Eq. (11). The relationship between the magnetic field $\mathbf {H}$ and $\mathbf {A}$ is expressed as follows:

14 $$\begin{aligned} \mathbf{{H}} = \frac{1}{\mu }\nabla \times \mathbf{{A}} \end{aligned}$$

After the outer boundary is discretized, it is assumed that one surface of the i element is at the air top interface and denoted by ${\Gamma _i}$. Combining Eq. (14) and adding the field source of MT through the second term in Eq. (11), the expression is shown as follows:

15 $$\begin{aligned} \int _{{\Gamma _i}} {\mathbf{{N}} \times \left( {\nabla \times {\varvec{{\tilde{A}}}}} \right) } \cdot \hat{n}dS = \int _{{\Gamma _i}} {\mathbf{{N}} \times \left( {\mu {\mathbf{{H}}_0}} \right) } \cdot \hat{n}dS \end{aligned}$$

The calculation area is subdivided into units of smaller subdomains. The vector and scalar potential of each element in the grid are represented by piecewise polynomial basis functions, and the expressions are listed as follows:

16 $$\begin{aligned}&{\varvec{{\tilde{A}}}} = \sum \nolimits _{j = 1}^{{n_{edge}}} {{{{\varvec{{\tilde{A}}}}}_j}{\mathbf{{N}}_j}} \end{aligned}$$

17 $$\begin{aligned}&\quad \tilde{\varphi }= \sum \nolimits _{j = 1}^{{n_{node}}} {{{\tilde{\varphi }}_j}{N_j}} \end{aligned}$$

18 $$\begin{aligned}&\quad \tilde{\lambda }= \sum \nolimits _{j = 1}^{{n_{node}}} {{{\tilde{\lambda }}_j}{N_j}} \end{aligned}$$

where ${{\mathbf{{N}}_j}}$ denotes the vector shape function on the $j{th}$ edge in the element, ${{N_j}}$ denotes the scalar shape function of the $j{th}$ node in the element, and ${{n_{edge}}}$ and ${{n_{node}}}$ represent the number of edges and nodes in the element, respectively. Combining Eqs. (11) to (13) and Eqs. (16) to (18), the matrix form of the system equations after finite element discretization is obtained as follows:

19 $$\begin{aligned}{}&\left( {\begin{array}{lll} {{\mathbf{{M}}_1} + i\omega \mu {\mathbf{{M}}_2}}&{}{\mu {\mathbf{{M}}_3}}&{}{{\mathbf{{M}}_4}}\\ {i\omega \mathbf{{M}}_3^T}&{}{{\mathbf{{M}}_5}}&{}\mathbf{{0}}\\ {\mathbf{{M}}_4^T}&{}\mathbf{{0}}&{}\mathbf{{0}} \end{array}} \right) \left( {\begin{array}{*{20}{c}} {{\varvec{{\tilde{A}}}}}\\ {\tilde{\phi }}\\ {\tilde{\lambda }} \end{array}} \right) = \left( {\begin{array}{l} {\mu \mathbf{{S}}}\\ 0\\ 0 \end{array}} \right) \end{aligned}$$

20 $$\begin{array}{*{20}l} {M_{{1,ij}} = \int_{\Omega } {\left( {\nabla \times {\mathbf{N}}_{i} } \right) \cdot \left( {\nabla \times {\mathbf{N}}_{j} } \right)d\Omega } ,} \hfill & {M_{{2,ij}} = \int_{\Omega } {\sigma {\mathbf{N}}_{i} \cdot {\mathbf{N}}_{j} d\Omega } } \hfill \\ {M_{{3,il}} = \int_{\Omega } {\sigma {\mathbf{N}}_{i} \cdot \nabla N_{l} d\Omega } ,} \hfill & {M_{{4,ik}} = - \int_{\Omega } {{\mathbf{N}}_{i} \cdot \nabla N_{k} d\Omega } } \hfill \\ {M_{{5,lk}} = \int_{\Omega } {\nabla N_{l} \cdot \sigma \nabla N_{k} d\Omega } ,} \hfill & {S_{i} = \int_{{\Gamma _{{air}} }} {{\mathbf{N}}_{i} \times H_{0} \cdot \mathop n\limits^{\frown } dS} } \hfill \\ \end{array}$$

where ${{\Gamma _{air}}}$ is the air top interface. i, j = 1, ..., ${{n_{edge}}}$ and l, k = 1, ..., ${{n_{node}}}$. We use the MUMPS direct solver to solve the $\mathbf {A}$-$\varphi$ system equations.

## FEM with a hybrid mesh

### Mesh generation

**Figure 1:**
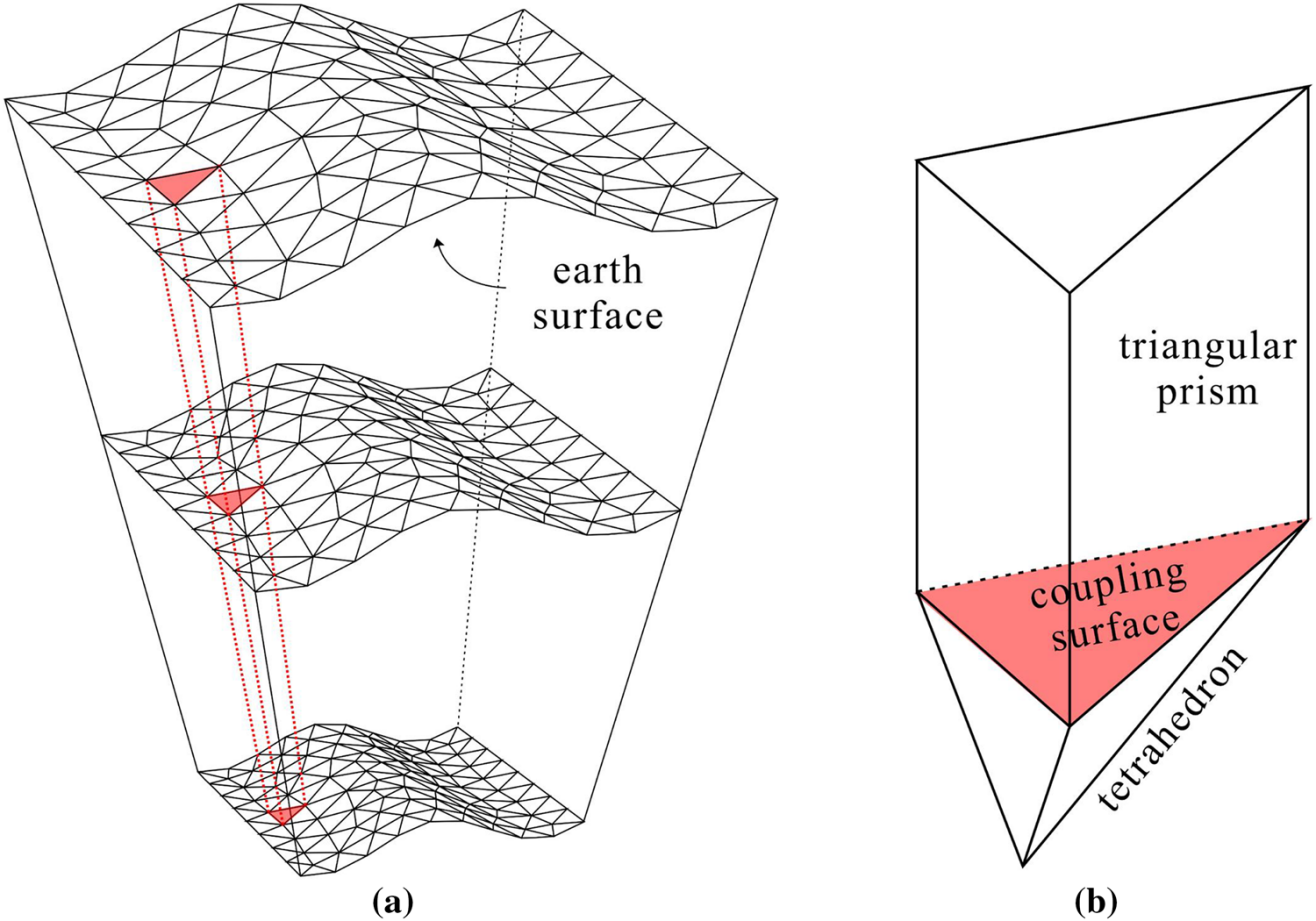
Meshing of a triangular prism element. (**a**) shows that two-layer triangular prism meshes can be directly spliced. The triangle at the top of the triangular prism can be generated with the topography. According to the top triangle, we extend the shape downward to create a triangular prism element. (**b**) represents the coupling of tetrahedral elements and triangular prism elements. The red surface is the coupling surface^35^.

The geoelectric model can be divided into near-surface areas and deep areas. The near-surface area is discretized by triangular prism elements, which can be seen as a boundary layer near the surface (Fig. 1a). The deep area is discretized by tetrahedrons, which lie below the boundary layer. The triangular prism and the tetrahedron can share a triangular face, which perfectly couples the triangular prism mesh and the tetrahedral mesh (Fig. 1b). We use the mesh generator in COMSOL to build the mesh, which is based on the Delaunay algorithm^28–33^. Other software capable of generating tetrahedrons, prisms, hexahedrons and other meshes can also be used to generate hybrid meshes, such as Gmsh^34^.

For the near-surface area of the geoelectric model, we use Delaunay triangulation algorithms to freely divide the Earth’s surface into triangular meshes and then extend the triangular meshes in the vertical direction to be a top layer comprised of triangular prisms. The extending distance is approximately 1 to 2 times the skin depth. The near-surface layer is approximately vertical. The change in the underground electromagnetic field can be measured by the skin depth. After passing our experiment, the vertical size of each subdivision layer is set to be less than 1/3 of the skin depth.

The areas adjacent to the triangular prism layers are the deep area below it and the air layer area above it. These adjacent areas are discretized into free tetrahedrons, which couple the upper and lower interfaces of the triangular prism layer through the triangular plane. The sizes of the tetrahedrons are based on the sizes of the triangles on the coupling surface and gradually grow away from the surface.

### Element analysis of triangular prism and pyramid

**Figure 2:**
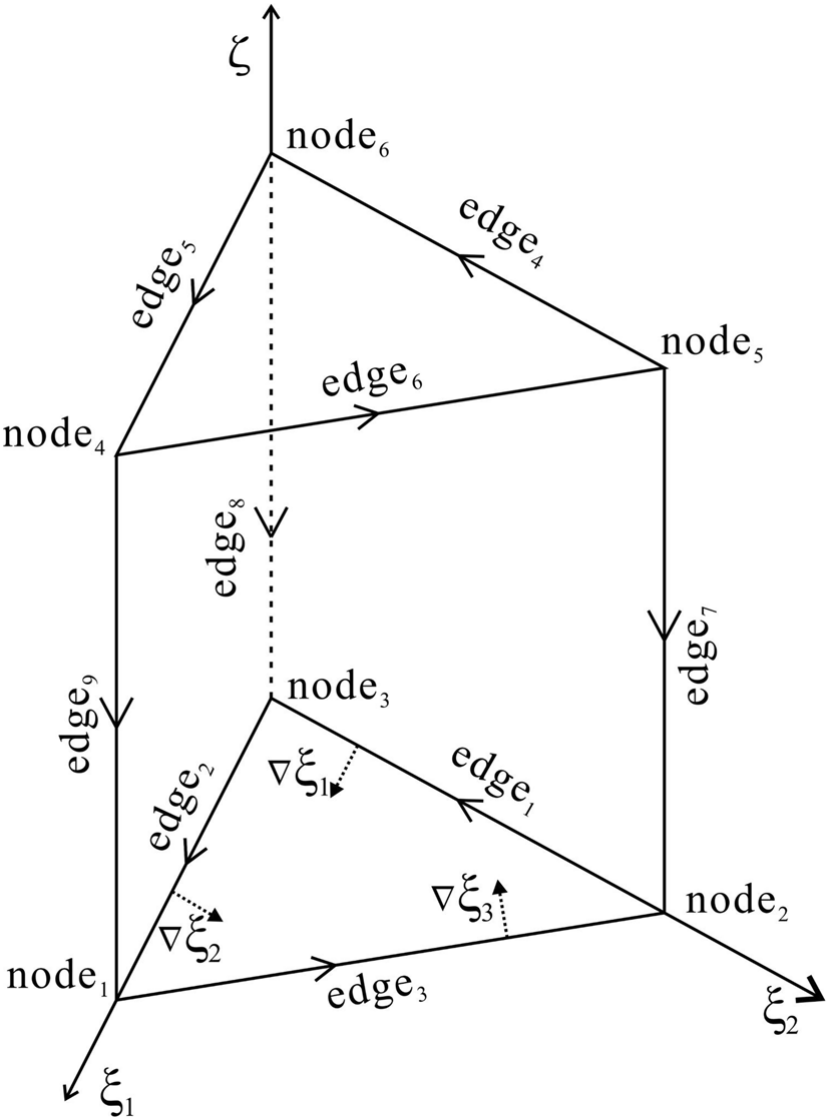
Illustration of one triangular prism element. There are six nodes and nine edges in the element, and the serial numbers of nodes and edges are defined in the coordinate system (${{\xi _1}}$, ${{\xi _2}}$, $\xi$).

The shape functions of the triangular prism elements (Fig. 2) consist of vector edge-shaped functions and scalar node-shaped functions^36^. A triangular prism element consists of two triangles at the top and the bottom and four rectangles around it. The shape functions of edges and nodes are shown in Appendix A.

**Figure 3:**
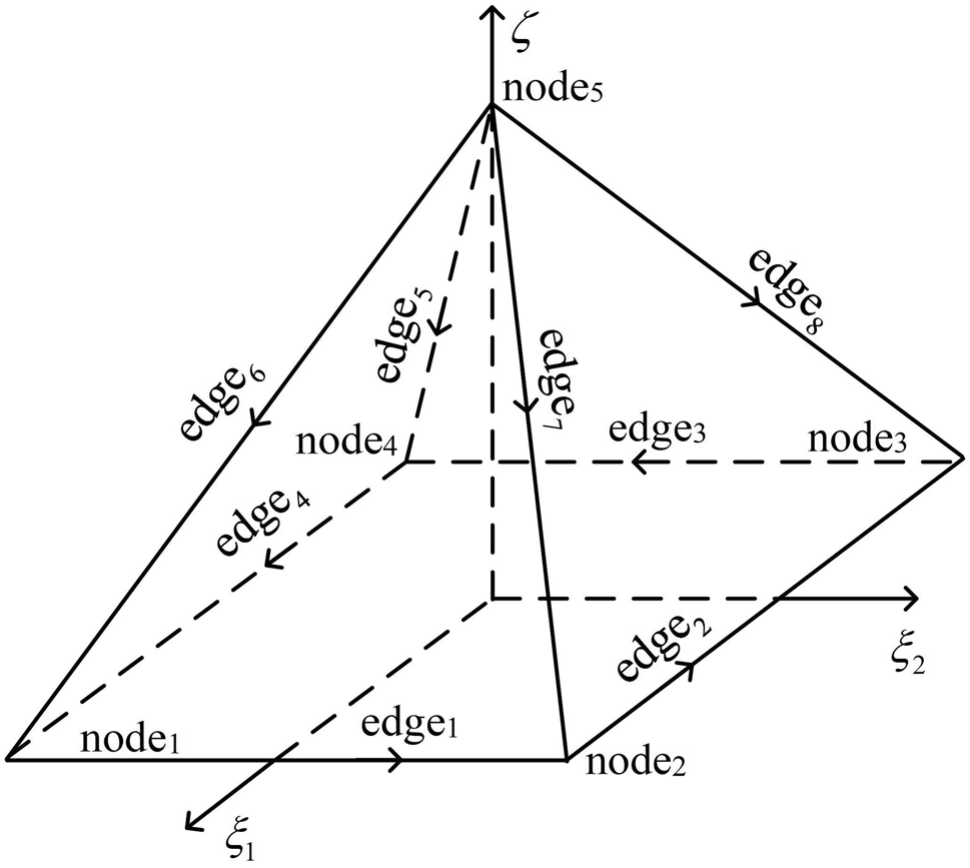
Illustration of one pyramid element. There are five nodes and eight edges in the element, and the serial numbers of nodes and edges are defined in the coordinate system (${{\xi _1}}$, ${{\xi _2}}$, $\xi$).

The shape functions of the pyramid elements (Fig. 3) consist of vector edge-shaped functions and scalar node-shaped functions. A pyramid prism element consists of one rectangle at the bottom and four triangles around it. The shape functions of the pyramid element are listed in Appendix A.

### Shape functions of second-order triangular prism element

**Figure 4:**
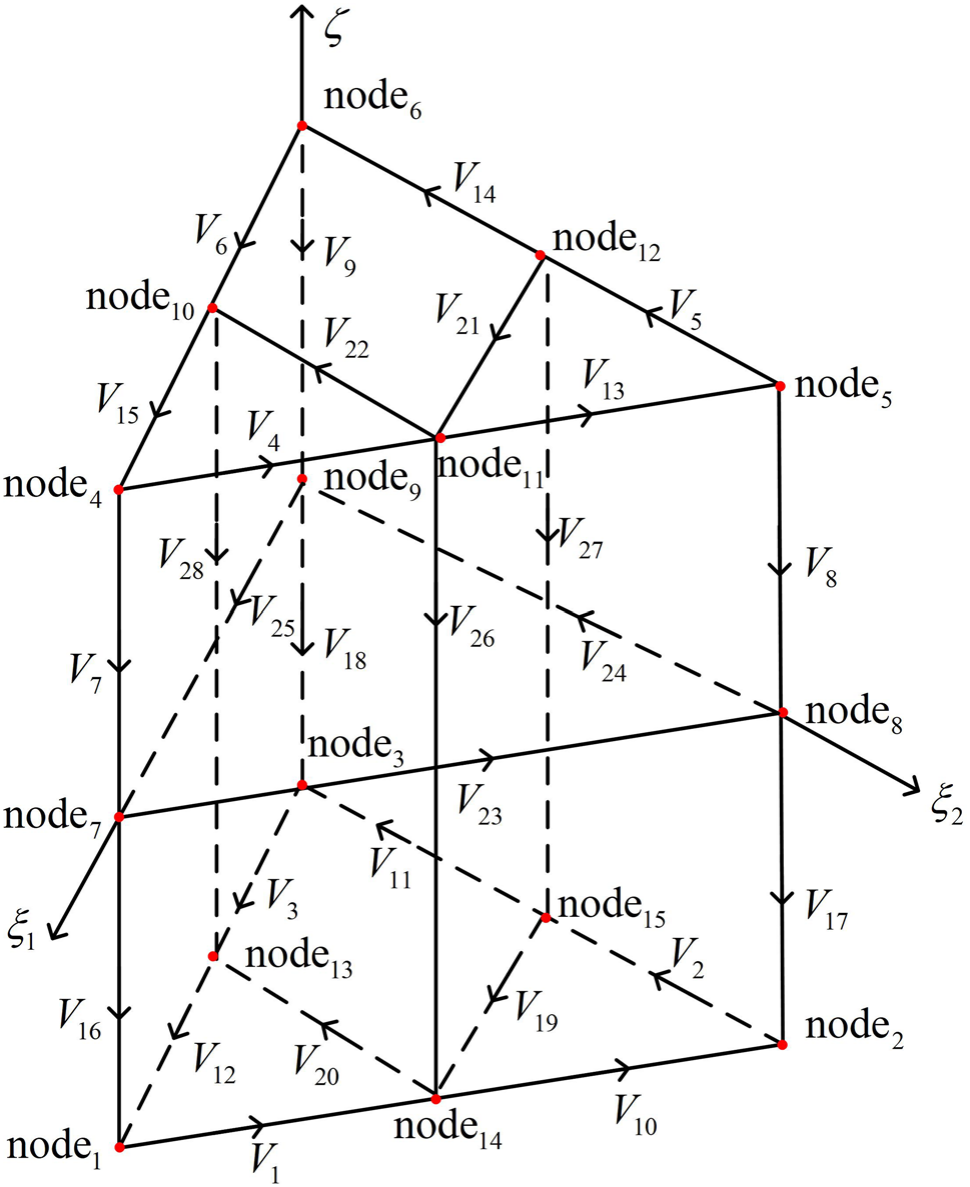
Illustration of one second-order triangular prism element; the serial numbers of nodes and edges are defined in the coordinate system (${{\xi _1}}$, ${{\xi _2}}$, $\xi$).

The quadratic triangular prism element is shown in Fig. 4. It has a total of 15 nodes, eighteen variables along edges and ten on faces in the element. V${_i}$ (i = 1, 2, ..., 28) is the variable in the element. The element illustrated in Fig. 4 has fifteen scalar node-based shape functions^37^.

The scalar node-based shape functions of the triangular prism element of corner nodes are:

21 $$\begin{aligned} \begin{array}{l} {N_i} = \frac{1}{2}{\xi _{{i_0}}}\left( {2{\xi _{{i_0}}} - 1} \right) \left( {1 + {\zeta _i}\zeta } \right) - \frac{1}{2}{\xi _i}\left( {1 - {\zeta ^2}} \right) , \\ {\zeta _i}\mathrm{{ = }}\left\{ {\begin{array}{*{20}{c}} 1&{}{i = 4, 5, 6}\\ { - 1}&{}{i = 1, 2, 3} \end{array}} \right. , {i_0}\mathrm{{ = }}\left\{ {\begin{array}{*{20}{c}} {i - 3}&{}{i = 4, 5, 6}\\ i&{}{i = 1, 2, 3} \end{array}} \right. \end{array} \end{aligned}$$

The scalar node-based shape functions of the mid-edge of the triangle are:

22 $$\begin{aligned} \begin{array}{l} {N_i} = 2{\xi _{{i_1}}}{\xi _{{i_2}}}\left( {1 + {\zeta _i}\zeta } \right) , {\zeta _i}\mathrm{{ = }}\left\{ {\begin{array}{*{20}{c}} 1&{}{i = 10, 11, 12}\\ { - 1}&{}{i = 13, 14, 15} \end{array}} \right. , \\ {i_1} = \left\{ {\begin{array}{*{20}{c}} 1&{}{i = 10, 11, 13, 14}\\ 2&{}{i = 12, 15} \end{array}} \right. , {i_2} = \left\{ {\begin{array}{*{20}{c}} 2&{}{i = 11, 14}\\ 3&{}{i = 10, 13, 12, 15} \end{array}} \right. \end{array} \end{aligned}$$

The scalar node-based shape functions of the mid-edge of the rectangle are:

23 $$\begin{aligned} {N_i} = {\xi _{{i_1}}}\left( {1 - {\zeta ^2}} \right) , {i_1} = i - 6, i = 7, 8, 9 \end{aligned}$$

Ahagon et al. presented that the vector edge-shaped functions are derived from those scalar node-based shape functions^38,39^. Their relationship is:

24 $$\begin{aligned} \sum \limits _i {{\mathbf{{N}}_{i,j}}} = \nabla {N_j} \end{aligned}$$

where the edge i-j is associated with the node j shape function. According to Eqs. 21–24, the 2nd vector edge-shaped functions of triangular prisms can be derived easily. Taking node 1, node 7 and node 11 as examples:

25 $$\begin{aligned}{}&\nabla {N_1} = \frac{1}{2}\left( {1 - \zeta } \right) \left( {4{\xi _1} - \zeta - 2} \right) \nabla {\xi _1} + \left( { - \xi _1^2 + \frac{1}{2}{\xi _1} + {\xi _1}\zeta } \right) \nabla \zeta \nonumber \\&\quad = \frac{1}{2}\left( {1 - \zeta } \right) \nonumber \\&\quad \left( {4{\xi _1} - \zeta - 2} \right) \left( {{\xi _2}\nabla {\xi _1} - {\xi _1}\nabla {\xi _2}} \right) \nonumber \\&\quad + \frac{1}{2}\left( {1 - \zeta } \right) \left( {4{\xi _1} - \zeta - 2} \right) \left( {{\xi _3}\nabla {\xi _1} - {\xi _1}\nabla {\xi _3}} \right) + \left( { - \xi _1^2 + \frac{1}{2}{\xi _1} + {\xi _1}\zeta } \right) \nabla \zeta \nonumber \\&\quad = \frac{1}{2}\left( {1 - \zeta } \right) \left( {4{\xi _1} - \zeta - 2} \right) {\mathbf{{W}}_{21}} + \frac{1}{2}\left( {1 - \zeta } \right) \left( {4{\xi _1} - \zeta - 2} \right) {\mathbf{{W}}_{31}} + \left( { - \xi _1^2 + \frac{1}{2}{\xi _1} + {\xi _1}\zeta } \right) \nabla \zeta \nonumber \\&\quad = {\mathbf{{N}}_{2,1}} + {\mathbf{{N}}_{13,1}} + {\mathbf{{N}}_{7,1}} \end{aligned}$$

26 $$\begin{aligned}{}&\quad \nabla {N_7} = \left( {1 - {\zeta ^2}} \right) \nabla {\xi _1} - 2\zeta {\xi _1}\nabla \zeta \nonumber \\&\quad = \left( {1 - {\zeta ^2}} \right) \left( {{\xi _2}\nabla {\xi _1} - {\xi _1}\nabla {\xi _2}} \right) + \left( {1 - {\zeta ^2}} \right) \left( {{\xi _3}\nabla {\xi _1} - {\xi _1}\nabla {\xi _3}} \right) \nonumber \\&\quad + \left( { - \xi _1^2 + \frac{1}{2}{\xi _1} - {\xi _1}\zeta } \right) \nabla \zeta + \left( {\xi _1^2 - \frac{1}{2}{\xi _1} - {\xi _1}\zeta } \right) \nabla \zeta \nonumber \\&\quad = \left( {1 - {\zeta ^2}} \right) {\mathbf{{W}}_{21}} + \left( {1 - {\zeta ^2}} \right) {\mathbf{{W}}_{31}} + \left( { - \xi _1^2 + \frac{1}{2}{\xi _1} - {\xi _1}\zeta } \right) \nabla \zeta \nonumber \\&\quad + \left( {\xi _1^2 - \frac{1}{2}{\xi _1} - {\xi _1}\zeta } \right) \nabla \zeta \nonumber \\&\quad = {\mathbf{{N}}_{8,7}} + {\mathbf{{N}}_{9,7}} + {\mathbf{{N}}_{4,7}} + {\mathbf{{N}}_{1,7}} \end{aligned}$$

27 $$\begin{aligned}{}&\quad \nabla {N_{11}} = 2{\xi _1}{\xi _2}\nabla \zeta + 2\left( {1 + \zeta } \right) {\xi _1}\nabla {\xi _2} \nonumber \\&\quad + 2\left( {1 + \zeta } \right) {\xi _2}\nabla {\xi _1} = 2{\xi _1}{\xi _2}\nabla \zeta + \frac{1}{2}\left( {1 + \zeta } \right) \left( {4{\xi _1} + \zeta - 2} \right) \left( {{\xi _1}\nabla {\xi _2} - {\xi _2}\nabla {\xi _1}} \right) \nonumber \\&\quad + \frac{1}{2}\left( {1 + \zeta } \right) \left( {4{\xi _2} + \zeta - 2} \right) \left( {{\xi _1}\nabla {\xi _2} - {\xi _2}\nabla {\xi _1}} \right) \nonumber \\&\quad + 2\left( {1 + \zeta } \right) {\xi _1}\left( {{\xi _3}\nabla {\xi _2} - {\xi _2}\nabla {\xi _3}} \right) \nonumber \\&\quad + 2\left( {1 + \zeta } \right) {\xi _2}\left( {{\xi _3}\nabla {\xi _1} - {\xi _1}\nabla {\xi _3}} \right) \nonumber \\&\quad = 2{\xi _1}{\xi _2}\nabla \zeta + \frac{1}{2}\left( {1 + \zeta } \right) \left( {4{\xi _1} + \zeta - 2} \right) {\mathbf{{W}}_{12}} \nonumber \\&\quad + \frac{1}{2}\left( {1 + \zeta } \right) \left( {4{\xi _2} + \zeta - 2} \right) {\mathbf{{W}}_{21}}\nonumber \\&\quad + 2\left( {1 + \zeta } \right) {\xi _1}{\mathbf{{W}}_{32}} + 2\left( {1 + \zeta } \right) {\xi _2}{\mathbf{{W}}_{31}}\nonumber \\&\quad = {\mathbf{{N}}_{14,11}} + {\mathbf{{N}}_{4,11}} + {\mathbf{{N}}_{5,11}} + {\mathbf{{N}}_{10,11}} + {\mathbf{{N}}_{12,11}} \end{aligned}$$

The other 2nd vector edge-shaped functions can be deduced in the same way as above. We can divide the vector edge-shaped functions into several categories as follows:

The vector edge-shaped functions for the bottom and top triangle edges can be expressed as:

28 $$\begin{aligned} \begin{array}{l} {\mathbf{{N}}_i}\mathrm{{ = }}\frac{1}{2}\left( {1 + {\zeta _i}\zeta } \right) \left( {4{\xi _{{i_2}}} + {\zeta _i}\zeta - 2} \right) {\mathbf{{W}}_{{i_1}{i_2}}},\\ {\zeta _i}\mathrm{{ = }}\left\{ {\begin{array}{*{20}{c}} 1&{}{i = 4, 5, 6, 13, 14, 15}\\ { - 1}&{}{i = 1, 2, 3, 10, 11, 12} \end{array}} \right. ,\\ {i_1} = \left\{ {\begin{array}{*{20}{c}} {1 }\\ {2 }\\ {3 } \end{array}\begin{array}{*{20}{c}} {i = 2, 3, 5, 6, }\\ {i = 1, 4, 11, 14}\\ {i = 10, 12, 13, 15 } \end{array}} \right. ,{i_2} = \left\{ {\begin{array}{*{20}{c}} {1 }\\ {2 }\\ {3 } \end{array}\begin{array}{*{20}{c}} {i = 1, 4, 7, 12, 15 }\\ {i = 2, 5, 8, 10, 13}\\ {i = 3, 6, 9, 11, 14} \end{array}} \right. \end{array} \end{aligned}$$

The vector edge-shaped functions for the rectangle edges (volume vector functions) can be expressed as:

29 $$\begin{aligned} \begin{array}{l} {\mathbf{{N}}_i}\mathrm{{ = }}\frac{1}{2}\left( {1 + {\zeta _i}\zeta } \right) \left( {{\zeta _i}\xi _{{i_2}}^2 - \frac{1}{2}{\zeta _i}{\xi _{{i_2}}} + {\xi _{{i_2}}}\zeta } \right) \nabla \zeta ,\\ {\zeta _i}\mathrm{{ = }}\left\{ {\begin{array}{*{20}{c}} 1&{}{i = 7, 8, 9}\\ { - 1}&{}{i = 16, 17, 18} \end{array}} \right. , {i_2} = \left\{ {\begin{array}{*{20}{c}} {1 }\\ {2 }\\ {3 } \end{array}\begin{array}{*{20}{c}} {i = 7, 16}\\ {i = 8, 17}\\ {i = 9, 18} \end{array}} \right. \end{array} \end{aligned}$$

The vector edge-shaped functions for the bottom and top triangle faces can be expressed as:

30 $$\begin{aligned} \begin{array}{l} {\mathbf{{N}}_i}\mathrm{{ = 2}}\left( {1 + {\zeta _i}\zeta } \right) {\xi _j}{\mathbf{{W}}_{{i_1}{i_2}}},\\ {\zeta _i}\mathrm{{ = }}\left\{ {\begin{array}{*{20}{c}} 1&{}{i = 7, 8, 9}\\ { - 1}&{}{i = 16, 17, 18} \end{array}} \right. , j = \left\{ {\begin{array}{*{20}{c}} 1&{}{i = 20, 22}\\ 2&{}{i = 19, 21} \end{array}} \right. ,\\ {i_1} = \left\{ {\begin{array}{*{20}{c}} 2&{}{i = 19, 20, 21, 22} \end{array}} \right. , {i_2} = \left\{ {\begin{array}{*{20}{c}} 1&{}{i = 19, 21}\\ 2&{}{i = 20, 22} \end{array}} \right. \end{array} \end{aligned}$$

The vector edge-shaped functions for the rectangle faces can be expressed as:

31 $$\begin{aligned} {\mathbf{{N}}_i}\mathrm{{ = }}\left( {1 - {\zeta ^2}} \right) {\mathbf{{W}}_{21}}, {i_1} = \left\{ {\begin{array}{*{20}{c}} 2&{}{i = 23}\\ 3&{}{i = 24, 25} \end{array}} \right. , {i_2} = \left\{ {\begin{array}{*{20}{c}} 1&{}{i = 23, 25}\\ 2&{}{i = 24} \end{array}} \right. \end{aligned}$$

32 $$\begin{aligned} {\mathbf{{N}}_i}\mathrm{{ = 2}}{\xi _{{i_1}}}{\xi _{{i_2}}}\nabla \zeta , {i_1} = \left\{ {\begin{array}{*{20}{c}} 1&{}{i = 26, 28}\\ 2&{}{i = 28} \end{array}} \right. , {i_2} = \left\{ {\begin{array}{*{20}{c}} 2&{}{i = 26}\\ 3&{}{i = 27, 28} \end{array}} \right. \end{aligned}$$

## Advantages of hybrid mesh

We use the numerical example of a layered model to illustrate the superiority of triangular prism elements in MT forward modeling with FEM. The apparent resistivity and phase are the responses, which help us to analyze the accuracy of the calculation.

To simplify the analysis, we use the highest frequency to calculate the smallest skin depth, which is used to stretch the triangular prism layer in the near-surface area. Although this size is sufficient for the calculation of an EM field with lower frequencies, it does not affect the analysis of the superiority of the triangular prism to the tetrahedron.

### Meshing the three-dimensional layered model

**Figure 5:**
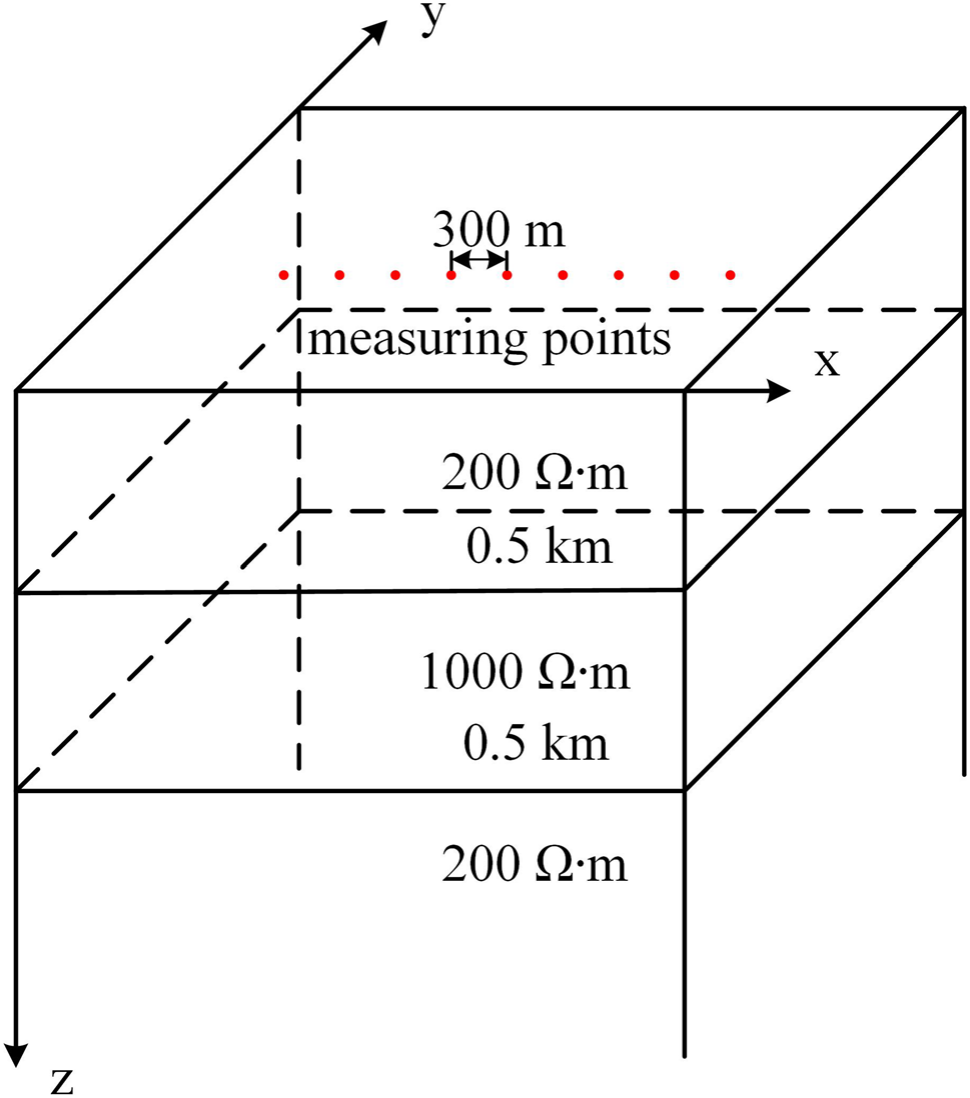
Three-dimensional layered geoelectric model.

The layered model is divided into three layers (Fig. 5). The resistivities of the first, second and third layers are 200 $\Omega \cdot$m, 1000 $\Omega \cdot$ m, and 200 $\Omega \cdot$m, respectively. The thicknesses of the first layer and the second layer are both 0.5 km. The measuring points are located from −1200 m to 1200 m along the X direction at Y = 0 m and Z = 0 m, with a distance of 300 m. The size of our entire calculation model is 20 km $\times$ 20 km $\times$ 70 km.

The sizes of mesh elements can be divided into horizontal sizes and vertical sizes and are controlled by the maximum unit size (MES) and growth rate. The control of these sizes mainly lies in the discretization of prismatic elements in the near-surface area. The size of the tetrahedron in the deep area depends on the size of the bottom surface of the triangular prism.

For the near-surface area, the horizontal sizes are controlled by the triangles used for meshing the ground. We use the triangular MES (TriMES) and triangular growth rate (TriGR) to generate these triangles. We use the prism growth rate (PriGR) to control the growth multiple of the vertical size of the triangular prism and control the total thickness of the prismatic layer.

For the deep area, the sizes of the top layer of the tetrahedrons, which are coplanar with the coupling plane, are controlled by the size of the coupling triangles. The sizes of the other tetrahedrons are controlled by the tetrahedral growth rate (TetGR).

**Table 1:** Parameters of different meshes for the three-dimensional layered model.l layered model.

	Mesh property	Horizontal size		Vertical size	
		TriMES (m)	TriGR	Thickness of the first layer (m)	PriGR/TetGR
Mesh A	Hybrid mesh	1500	1.3	8	1.3/1.5
Mesh B1	Tetrahedral mesh	1500	1.3	500	1.5
Mesh B2		250	1.3	200	1.5
Mesh B3		100	1.3	65	1.5

MES means maximum unit size. The horizontal size is controlled by the triangular MES (TriMES) at the Earth’s surface and triangular growth rate (TriGR). The TriGR represents the maximum element growth rate of the triangular element size. Vertical size is controlled by the thickness of the prismatic layer and tetrahedral growth rate (TetGR). The TetGR represents the maximum element growth rate of the tetrahedral element size. The prismatic growth rate (PriGR) represents the vertical prismatic element growth rate of the prism layer thickness

We use four types of meshes (Table 1) to discretize the three-dimensional layered model. Mesh A is a hybrid mesh that is divided into two parts: the upper part is a triangular prism mesh, and the lower part is a tetrahedral mesh. The horizontal size is determined by the size of the triangular element at the top. The TriMES is 1500 m, and the TriGR is 1.3. The vertical size of the hybrid mesh is determined by the vertical size of the triangular prism and the tetrahedron at the same time. The vertical size of the first prismatic layer is 8 m, and the PriGR is equal to the TetGR. The total number of prismatic layers is 10. Below the prismatic layers, the vertical size of the tetrahedral element is controlled by the TriMES and TetGR. The TetGR in hybrid mesh A is 1.5.

**Figure 6:**
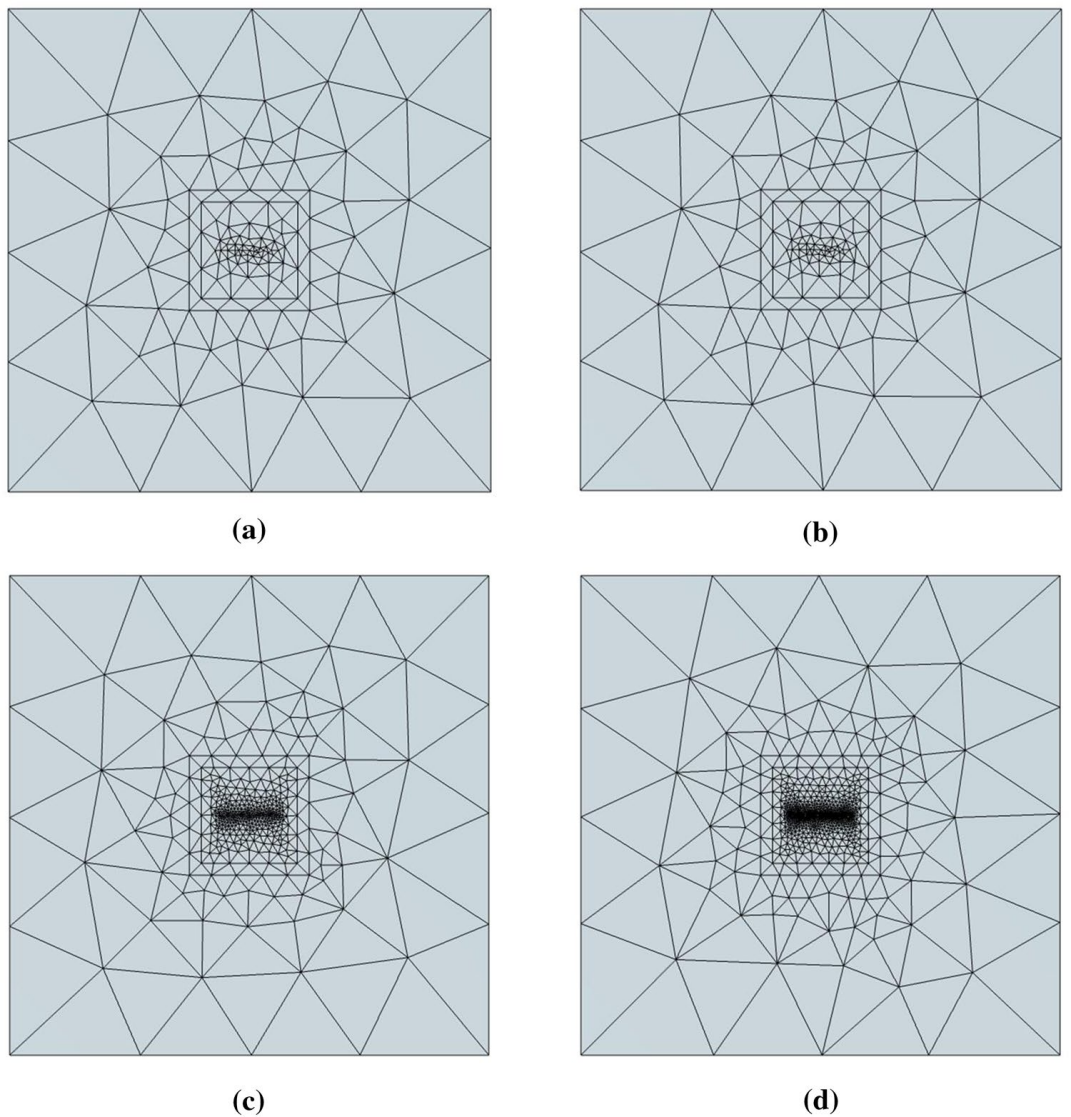
Mesh refinement at measuring points of the three-dimensional layered geoelectric model. (**a**) is the mesh refinement at the measuring points of Mesh A. (**b**) is the mesh refinement at the measuring points of Mesh B1. (**c**) is the mesh refinement at the measuring points of Mesh B2. (**d**) is the mesh refinement at the measuring points of Mesh B3^40^.

**Figure 7:**
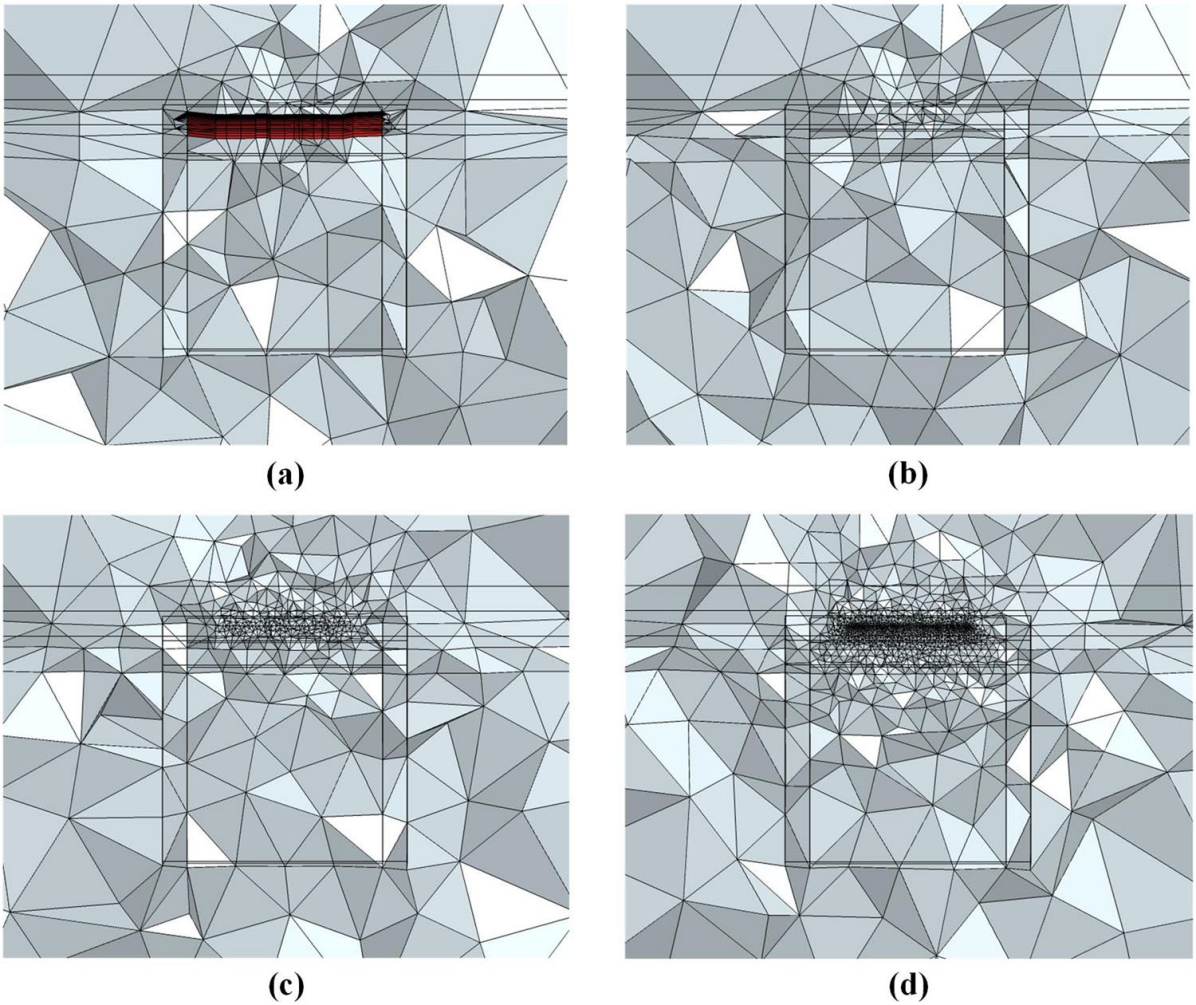
Grid diagram of the cross section of the three-dimensional layered geoelectric model. (**a**) is the cross section of Mesh A. The red part represents the triangular prism mesh, and the remaining part represents the tetrahedral mesh. (**b**) is the cross section of Mesh B1. (**c**) is the cross section of Mesh B2. (**d**) is the cross section of Mesh B3^40^.

Meshes B1, B2, and B3 are tetrahedral meshes, and their granularities are different. Mesh B1 is the coarsest-grained mesh. The horizontal size of the mesh B1 element is the same as that of the triangular prism element, and the TriMES is 1500 m. The vertical size of the mesh B1 element in the first layer is approximately 500 m under the premise of effective meshing for good mesh quality. Meshes B2 and B3 have smaller granularity than mesh B1. The TriMES and the first layer thickness of the B2 mesh are 250 m and approximately 200 m, respectively. The TriMES and the first layer thickness of the B2 mesh are 100 m and approximately 65 m, respectively. We compared the mesh refinement at measuring points (Fig. 6) and the grid diagram of the cross section (Fig. 7) of the four grids.

### Higher-order shape function

**Figure 8:**
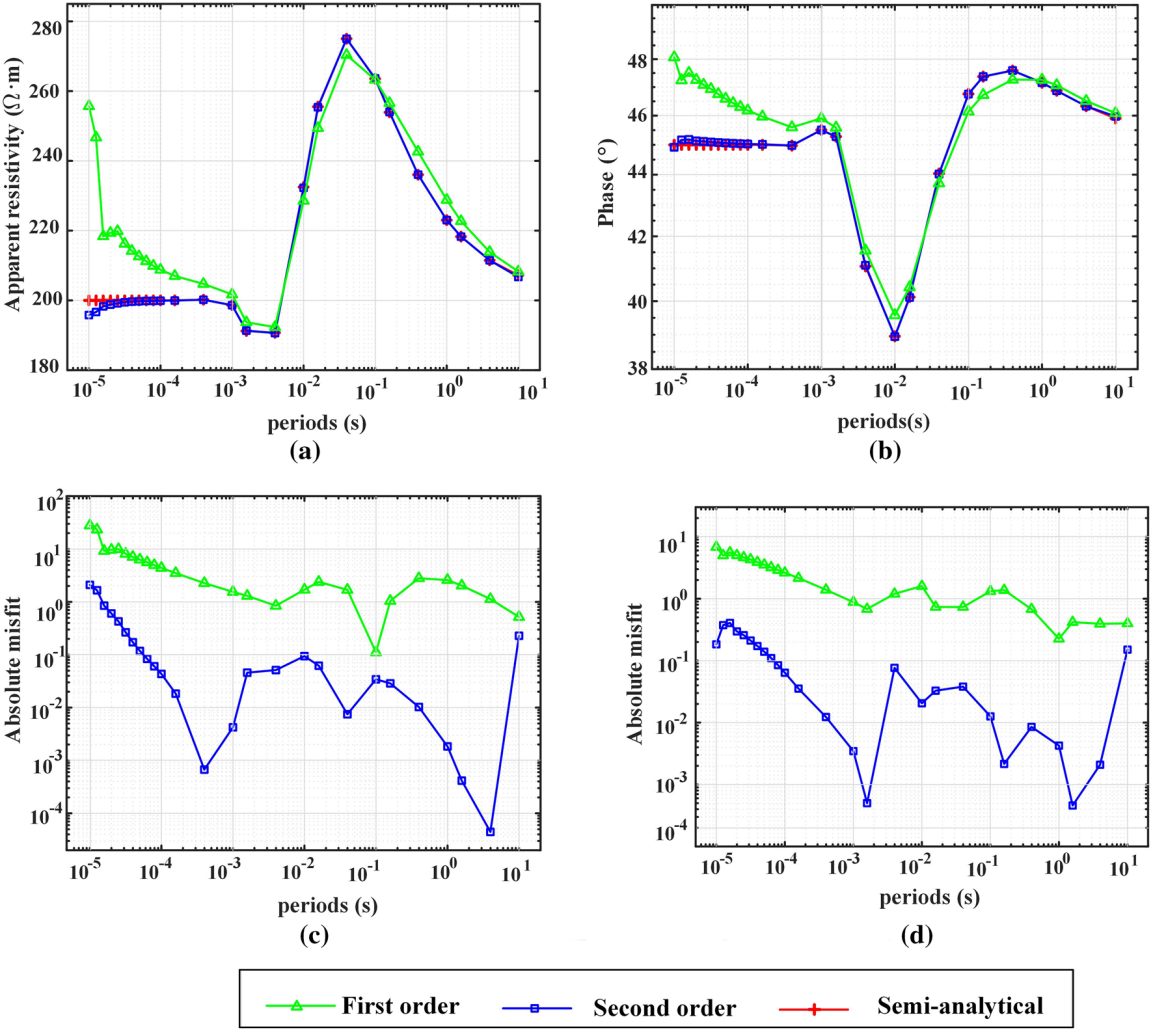
The MT responses of the three-dimensional layered model calculated with hybrid meshes of different orders. Apparent resistivity and phases are shown in (**a**) and (**b**), respectively. The misfits of the apparent resistivity and phases are shown in (**c**) and (**d**), respectively. The absolute misfit is normalized by the semianalytic solution.

MT responses are calculated in the frequency range of 1E5 Hz to 1E−1 Hz with hybrid mesh^20^ of different orders. In total, 26 frequency points were calculated, of which 10 frequency points were equally spaced between 1E5 Hz and 1E4 Hz, and the other frequency points were equally spaced from 1E4 Hz to 1E−1 Hz. The apparent resistivity and phase change with frequency are shown in Fig. 8. When the shape function is first order, the maximum absolute errors of the apparent resistivity and phase are 27.8510% and 6.8170%, respectively. When the shape function is second order, the maximum absolute errors of the apparent resistivity and phase are 2.0968% and 0.4063%, respectively. All the computations are implemented in MATLAB 2019 with an Intel Core i5-10400 CPU @ 2.90 GHz and 32 GB RAM.

### Mesh quality

**Figure 9:**
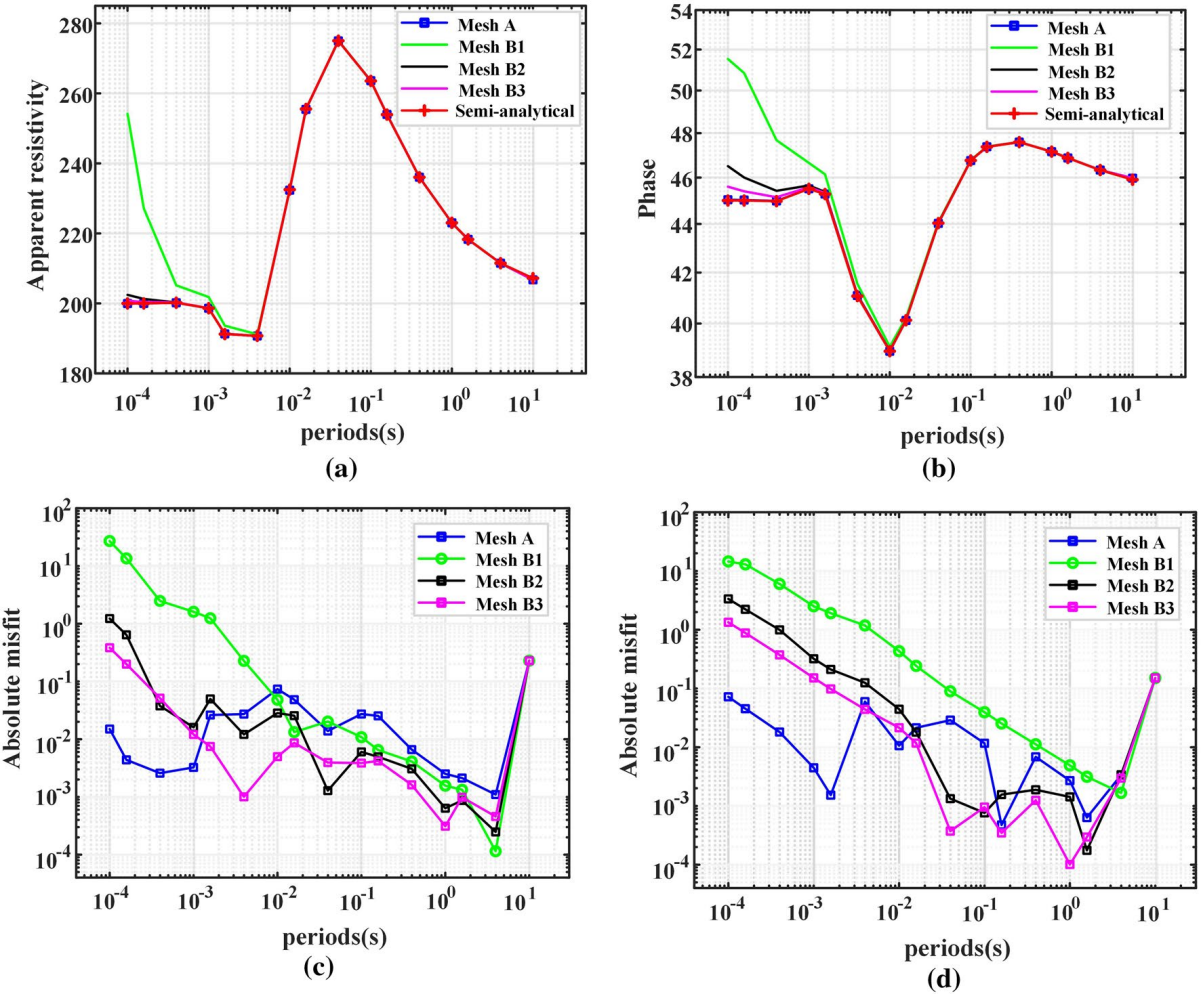
The MT responses of the three-dimensional layered model calculated with different meshes. Apparent resistivities and phases are shown in (**a**) and (**b**), respectively. The misfits of the apparent resistivities and phases are shown in (**c**) and (**d**), respectively. The absolute misfit is normalized by the semianalytic solution.

MT responses are calculated in the frequency range of 1E4 Hz to 1E−1 Hz. The apparent resistivity and phase change with frequency are shown in Fig. 9. The accuracy of the apparent resistivity and phase calculated by different meshes is quite different in this frequency range. We use the root mean square error (RMSE) to measure the overall error of the apparent resistivity and phase, and the reference solution uses the semianalytical solution of the 1D layered model.

For a mesh containing only tetrahedrons, the smaller the mesh granularity is, the smaller the calculated response misfit. For the layered model, the vertical size of the element is a key factor affecting the calculation accuracy. Considering the element quality, the horizontal size of the element needs to be consistent with its vertical size. However, this causes the number of elements (NE) and number of DoFs to increase sharply, and the calculation time increases with additional DoFs.

A hybrid mesh with a triangular prism can overcome this limitation. Even if the aspect ratio of the triangular prism element is greater than 100 times, high accuracy can be obtained. When the accuracy is not limited by the aspect ratio, the number of required elements is greatly reduced, and the required DoF number is also reduced. The DoF, mean absolute error (MAE) misfit and NE of the response results are all shown in Table 2.

**Table 2:** Calculation results of different meshes for the three-dimensional layered model.

	Mesh property	NE	DoF	NNZ	Time (min)	MAE ($\%$)	
						Apparent resistivity	Phase
Mesh A	Hybrid mesh (First order)	11,014	18,234	555,213	1.783	5.462	2.371
	Hybrid mesh (Second order)	11,014	112,082	8,786,634	8.833	**0.2679**	**0.104**
Mesh B1	Tetrahedral mesh	10,079	81,616	5,101,464	5.25	62.396	19.412
Mesh B2		185,593	1,439,313	92,848,086	42.133	2.765	3.802
Mesh B3		1,235,632	9,513,920	616,910,456	548.15	**0.966**	**2.143**

The number of element (NE) represents the total number of all elements in a hybrid or tetrahedral mesh. The DoF number and number of nonzeros (NNZ) reflect the basic situation of the FE system matrix. Based on the semianalytic solution, we use the MAE to measure the misfit of the results generated from different meshes. We provide the memory occupied by the four meshing calculations

Significant values are in [bold].

### System matrix quality

Table 2 shows the comparison of the FE system element number, DoF, calculation time and numerical solution accuracy information of different grids. A higher order is helpful to improve the accuracy of the numerical solution for the prismatic element. A comparison of the calculation results of mesh A and mesh B1 shows that mesh A has a greater DoF and more accurate numerical solutions. A comparison of the calculation results of meshes A and B2 shows that even if the DoF number of the tetrahedral mesh is eight times that of the hybrid mesh, the accuracy of the hybrid mesh is much higher than that of the tetrahedral mesh. A comparison of the results of meshes A and B3 shows that when the DoF number of the tetrahedral mesh is 56 times that of the hybrid mesh, the accuracy of the tetrahedral mesh can be at the same level as that of the hybrid mesh. This shows that the mesh type not only affects the size and DoF number of the system matrix but may also affect the solution properties of the system matrix.

The sparsity distribution of the system matrix of different meshes can reflect that the system matrix of the hybrid grid has better solution properties. For tetrahedral grids, as the number of DoFs increases, the bandwidth of the system matrix gradually weakens. The DoF of the hybrid grid is between the number of DoFs of meshes B1 and B2, but the bandwidth of its system matrix is notably better than that of the other two tetrahedral meshes.

## Numerical example: DTM1

**Figure 10:**
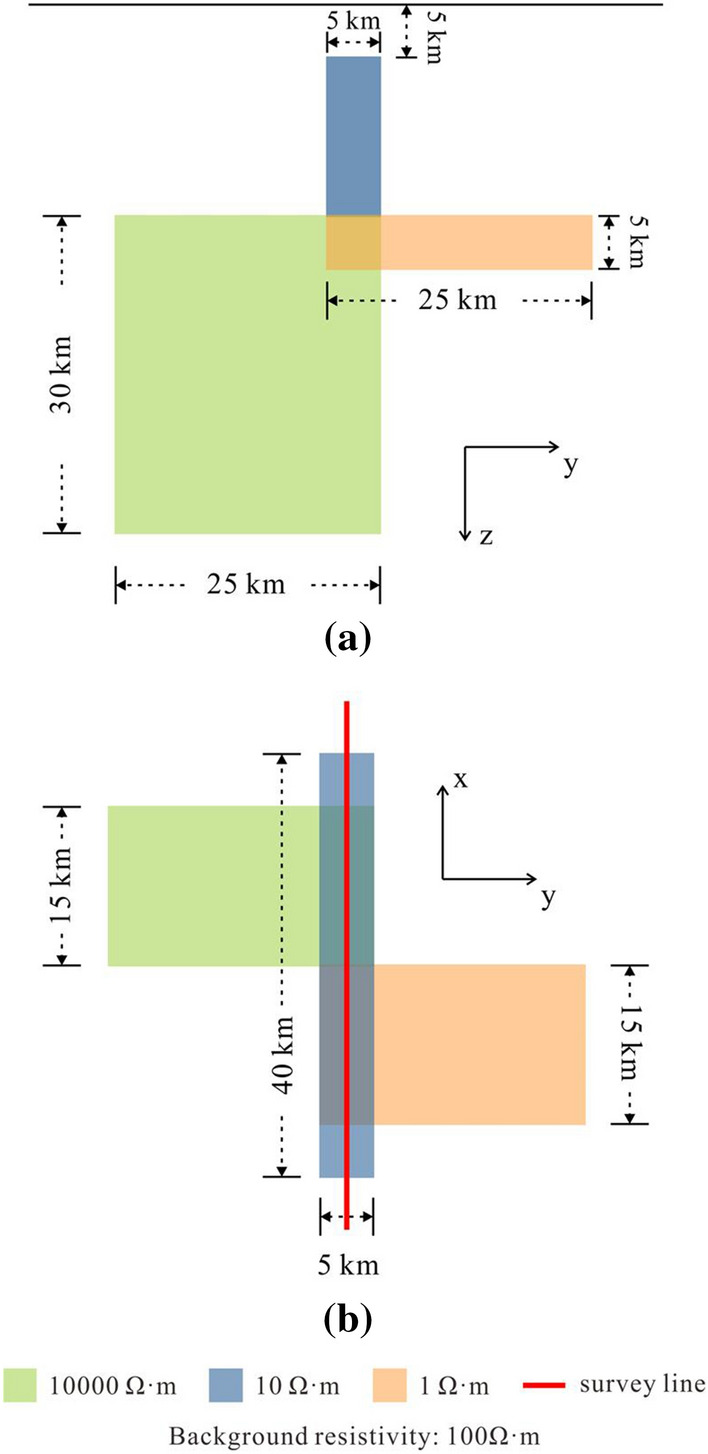
DTM1 model. (**a**) and (**b**) represent the vertical and horizontal sections of the model, respectively. The survey line is shown in the horizontal section.

The hybrid mesh mainly uses triangular prism elements to process near-surface areas, which are often related to the calculation accuracy of high-frequency data. To verify the role of the hybrid mesh in improving the accuracy of the high-frequency data solution, we increase the complexity of the model on the basis of the three-dimensional layered model and select the DTM1 model^41–44^ to show the advantages of the prism elements. A survey line is selected to record the responses of apparent resistivity and phase (Fig. 10). In the public DTM1 test data set, the results of four often used methods, Han’s^44^, Mackie’s^42^, wsinv3dmt^41^, and mt3dinv^45^, are summarized. We compare the results of these four methods.

We set up four different meshes in total, namely, a hybrid mesh and three tetrahedral meshes. The parameters of different meshes are shown in Table 3. In the hybrid mesh, the TriMES, which controls the size of the horizontal element, is set to 2000 m, and the thickness of the first layer of the triangular prism element is 318 m. In the tetrahedral mesh, the TriMES gradually changes from 2000 m to 500 m. The size of the tetrahedral element on the first layer is approximately equal to the TriMES on the surface. In the four meshes, the growth rate of both the triangular element and the tetrahedral element, TriGR and TetGR, is set to 1.5. The system equations established by TriMES’s 1000 m tetrahedral mesh and TriMES’s 2000 m hybrid mesh have the same DoF. The tetrahedral mesh of 500 m TriMES can be regarded as a dense tetrahedral mesh.

**Table 3:** Parameters of different meshes for DTM1 model.

Mesh property	Relationship with hybrid grid	Horizontal size		Vertical size		NE	DoF	Time (min)	Memory (GB)
		TriMES (m)	TriGR	Thickness of the first layer (m)	PriGR/TetGR				
Hybrid mesh		2000	1.5	318	1.5	34,489	350,809	11.833	33.04
Tetrahedral mesh	Similar horizontal size with that in hybrid mesh	2000	1.5	2000	1.5	22,083	170,129	3.467	15.383
	Similar DoF with that in hybrid mesh	1000	1.5	1000	1.5	46,340	356,659	8.933	29.27
	Much denser than other meshes	500	1.5	500	1.5	153,754	1,180,702	55.833	109.87

**Figure 11:**
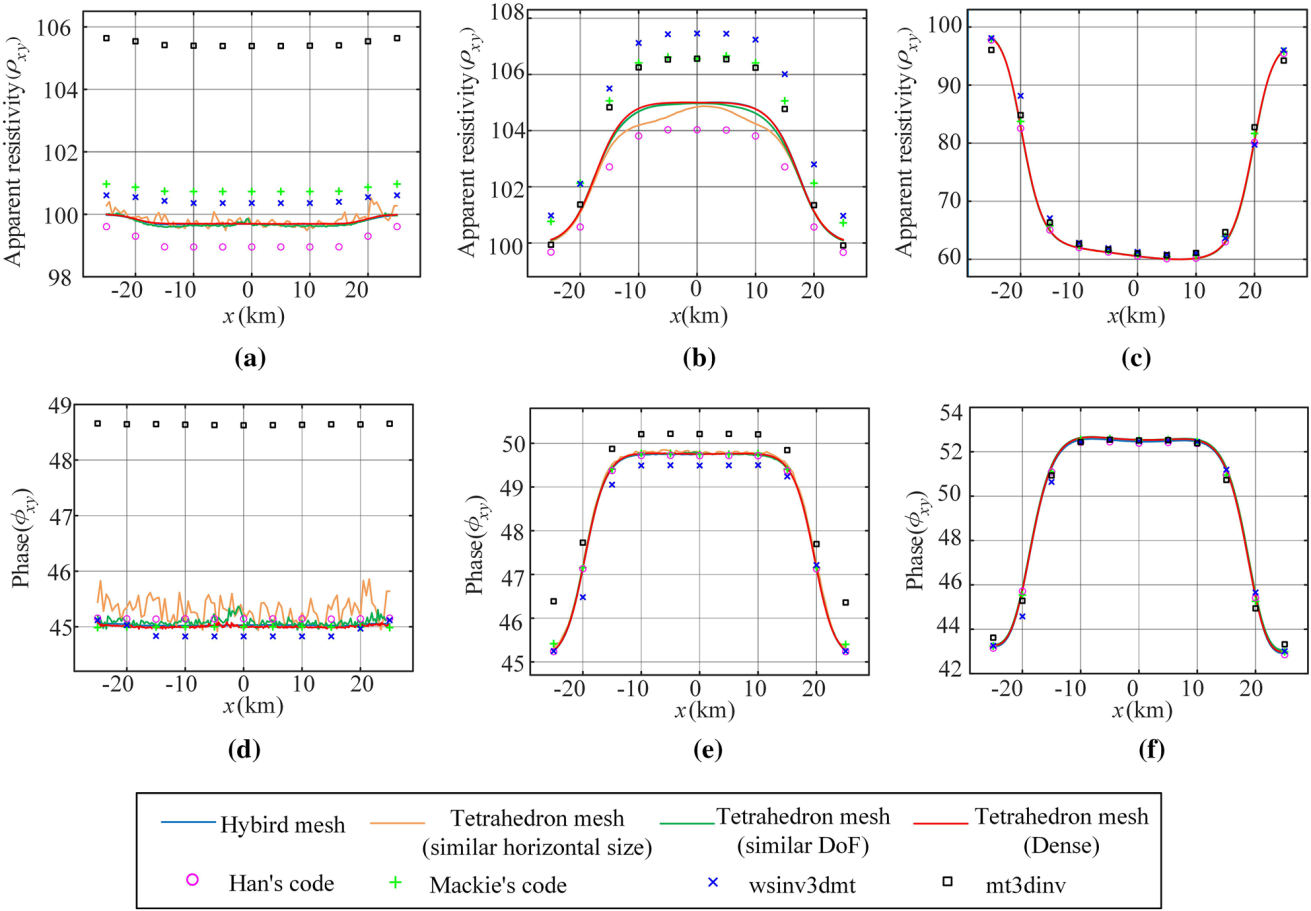
Calculation results of the DTM1 model. (**a**) and (**d**) represent the apparent resistivity result and phase result at a frequency of 10 Hz, (**b**) and (**e**) represent the apparent resistivity result and phase result at a frequency of 1 Hz, and (**c**) and (**f**) represent the apparent resistivity result and phase result at a frequency of 0.1 Hz. The responses of Nam’s code are obtained with FEM^43^ by Han/Lee^44^. The responses of Mackie’s code are obtained using the finite difference method by Mackie et al.^41^. The responses of wsinv3dmt^42^ and mt3dinv are obtained by the FD codes^45^.

The calculation results of the apparent resistivity and phase at frequencies of 0.1 Hz, 1 Hz, and 10 Hz are shown in Fig. 11. When the frequency is 10 Hz (Fig. 11a, d), the denser the tetrahedral mesh is, the smaller the oscillation of the apparent resistivity curve and the phase curve, but neither is as smooth as the response curve of the hybrid mesh. When the frequency is 1 Hz (Fig. 11b, e), the oscillation of the response curves of the tetrahedral mesh is reduced, but there is still a gap between the smoothness of the response curve of the hybrid mesh. Up to the frequency of 0.1 Hz (Fig. 11c, f), the smoothness of the response curves of the tetrahedral mesh is equivalent to that of the hybrid mesh. When calculating the MT high-frequency electromagnetic field, the discrete equation using the hybrid mesh is smoother, and the calculation result has higher accuracy.

When the horizontal size of the hybrid mesh and the tetrahedral mesh are both approximately 2000 m, because the triangular prism element in the hybrid mesh can reduce the vertical size without reducing the mesh quality, the hybrid mesh has more DoFs and smoother responses. When we refine the tetrahedral mesh, we reduce its horizontal size to approximately 1000 m and set the vertical size to approximately 1000 m to ensure that the number of DoFs of the tetrahedral mesh is similar to that of the hybrid mesh. The apparent resistivity and phase curves calculated by this dense tetrahedral mesh still oscillate significantly. The tetrahedral mesh is further refined, and the horizontal and vertical sizes are both set to approximately 500 m. At this time, the number of DoFs is nearly twice that of the hybrid mesh, but the calculation result is still not as stable. This shows that the hybrid mesh is indeed more efficient than the pure tetrahedral mesh in calculating high-frequency data.

## Numerical example: sloped terrain models

**Figure 12:**
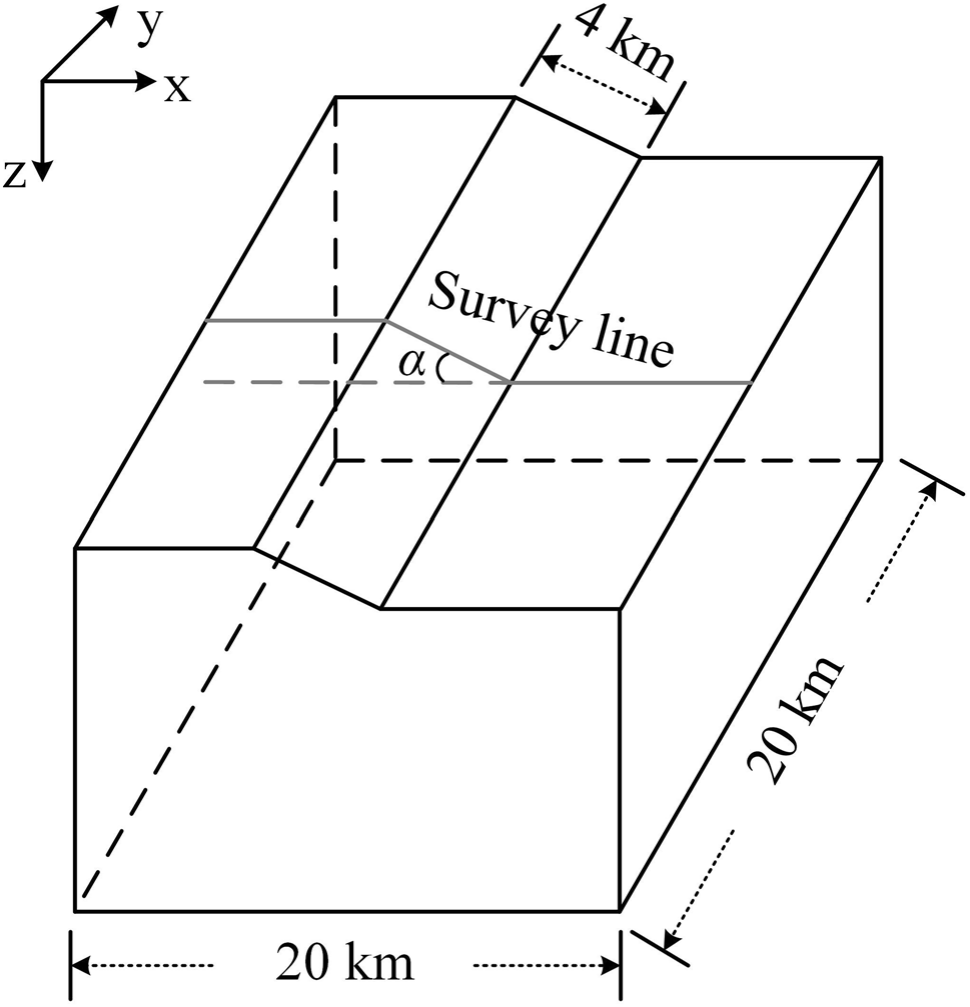
Sloped terrain models. The tilt angles of the slope are 10, 20, and 30 degrees.

Due to the volume effect of the electromagnetic method, the calculation of the high-frequency electromagnetic response is more difficult when the terrain is undulating. We adjust the change in the terrain undulation angle to analyze the improvement in the calculation efficiency of the hybrid mesh under different terrain conditions when the frequency is 1E4 Hz (Fig. 12).

**Table 4:** Parameters of different meshes for the sloped terrain models.

Tilt angle	Mesh property	Horizontal size		Vertical size		NE	DoF	Time (min)	Memory (GB)
		TriMES (m)	TriGR	Thickness of the first layer (m)	PriGR/TetGR				
10	Hybrid mesh	120	1.5	15	1.0/1.5	39,112	402,554	6.7	40.9
	General tetrahedral mesh	120	1.5	120	1.5	53,403	414,966	6.483	41.62
	Dense tetrahedral mesh	50	1.5	50	1.5	101,733	786,072	8.683	62.7
20	Hybrid mesh	120	1.5	15	1.5	37,873	394,195	6.417	40.07
	General tetrahedral mesh	120	1.5	120	1.5	55,327	430,114	7.567	43.07
	Dense tetrahedral mesh	50	1.5	50	1.5	112,294	867,347	10.483	71.2
30	Hybrid mesh	120	1.5	15	1.5	35,659	350,706	4.467	32.63
	General tetrahedral mesh	120	1.5	120	1.5	65,500	508,124	9.867	55.29
	Dense tetrahedral mesh	50	1.5	50	1.5	116,996	903,361	12.817	79.18

We set up three kinds of meshes, namely, a hybrid mesh, general tetrahedral mesh and dense tetrahedral mesh. The parameters of different meshes are shown in Table 4. The hybrid mesh and general tetrahedral mesh have the same horizontal element size, and their TriMESs are both 120 m. The horizontal element size of the dense tetrahedral mesh is smaller, and its TriMES is 50 m. The thickness of the first layer of triangular prism elements in the hybrid grid is 15 m. The vertical size of the first tetrahedron in the other two tetrahedral meshes is approximately equal to its horizontal size. Among these meshes, the mesh growth rates of triangles and tetrahedrons are both 1.5. The system matrix produced by the hybrid mesh and the tetrahedral mesh with the same horizontal element size has a similar number of DoFs, while the system matrix produced by the tetrahedral grid with the smallest horizontal element size has nearly twice the number of DoFs.

**Figure 13:**
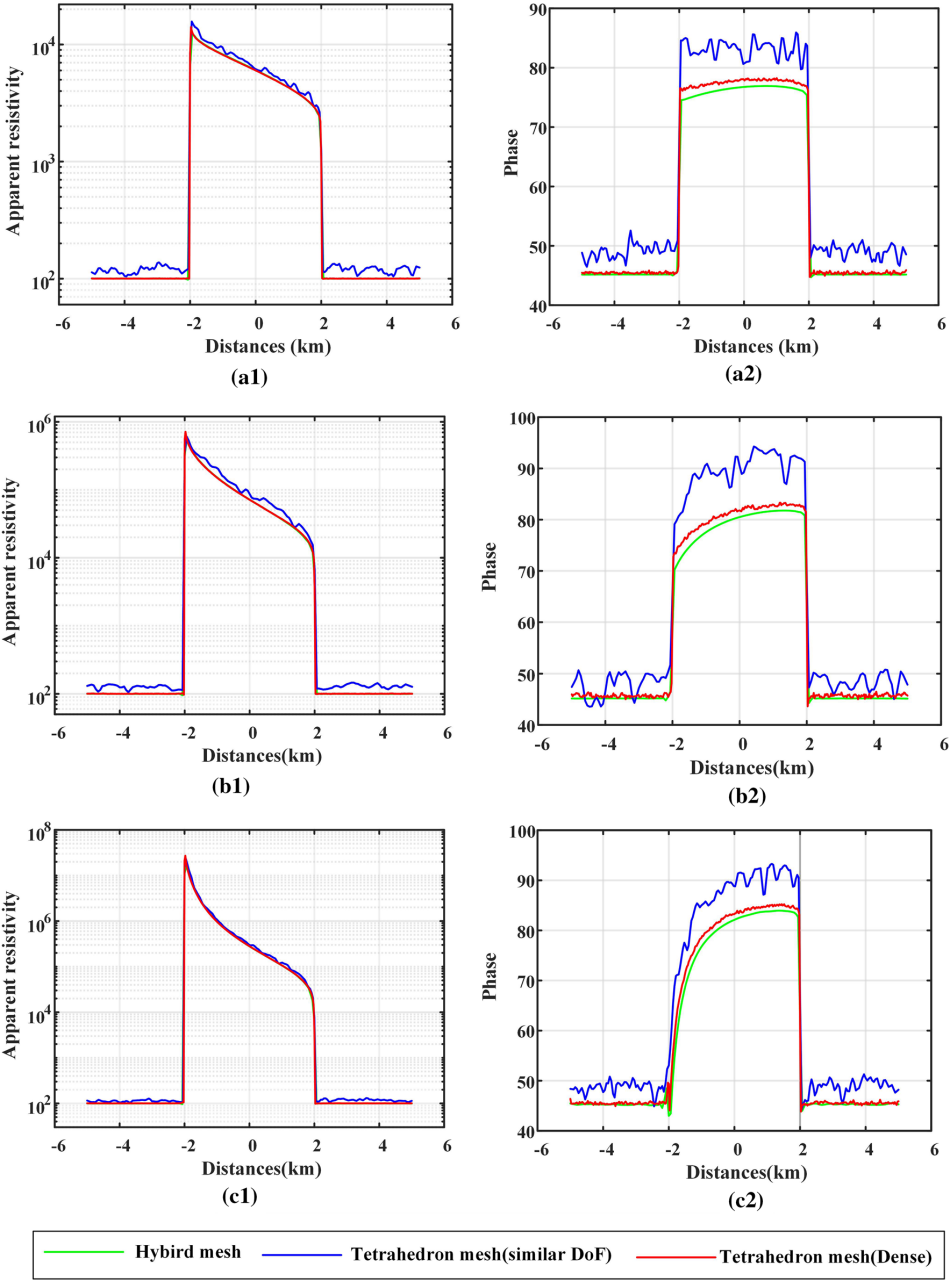
For the sloped terrain model, when the tilt angle changes, the MT response results of the xy component are calculated by different meshes. (**a**1), (**b**1) and (**c**1) represent the calculation results of the apparent resistivity, and (**a**2), (**b**2) and (**c**2) represent the calculation results of the phase. (**a**), (**b**) and (**c**) represent the calculation results when the tilt angle is 10, 20, and 30 degrees, respectively.

**Figure 14:**
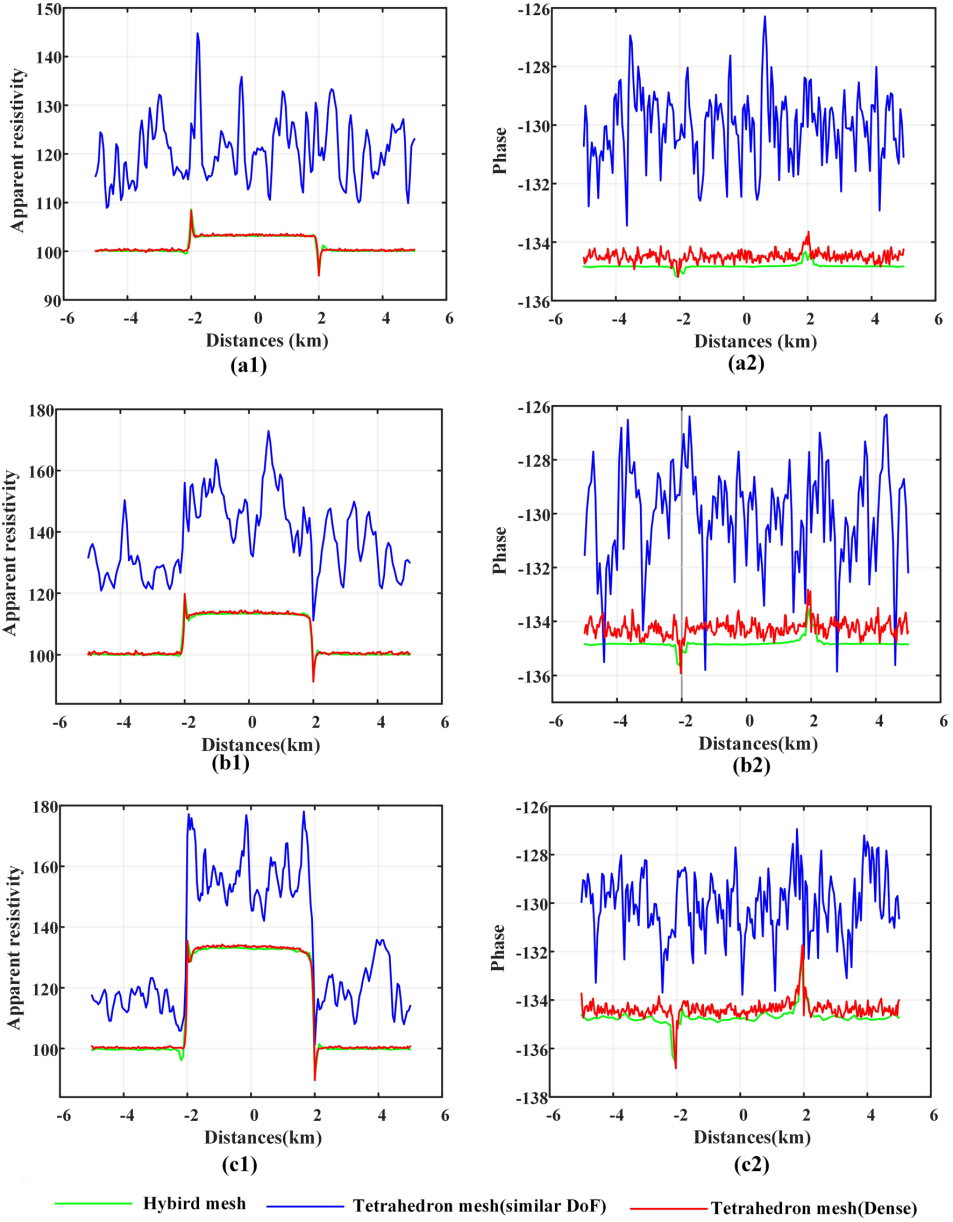
For the sloped terrain model, when the tilt angle changes, the MT response results of the yx component are calculated by different meshes. (**a**1), (**b**1) and (**c**1) represent the calculation results of the apparent resistivity, and (**a**2), (**b**2) and (**c**2) represent the calculation results of the phase. (**a**), (**b**) and (**c**) represent the calculation results when the tilt angle is 10, 20, and 30 degrees, respectively.

When the tilt angle changed, we calculated the responses of the xy component (Fig. 13) and the responses of the yx component (Fig. 14) with various types of meshes. For the xy component (Fig. 13), the accuracy of the resolution of the apparent resistivity accuracy is similar, and the calculation results of the general tetrahedral mesh have weak oscillations. The difference in the calculation results of different meshes is obvious in the phase results, but the difference in oscillating properties is essentially unchanged with the change in the tilt angle. Note that for both the apparent resistivity result and the phase result, the calculation result of the hybrid mesh is very stable for different tilt angle models.

The calculation results of the yx component are consistent with those of the xy component at three points. (1) the calculation results oscillate, but the hybrid mesh calculation results oscillate the least; (2) the oscillating difference of the results calculated by different meshes hardly changes with the change in tilt angle of sloped terrain models; and (3) the difference of oscillating is more obvious in the phase results.

The inconsistent point between the yx component and the xy component is that the response of the yx component has more dramatic oscillation (by comparing Figs. 13 and 14). The elevation on the slope changes along the X direction, which brings greater misfit to the yx component. It can be suggested that there may be a large misfit in the direction of large topographic relief.

## Numerical example: terrain relief model

**Figure 15:**
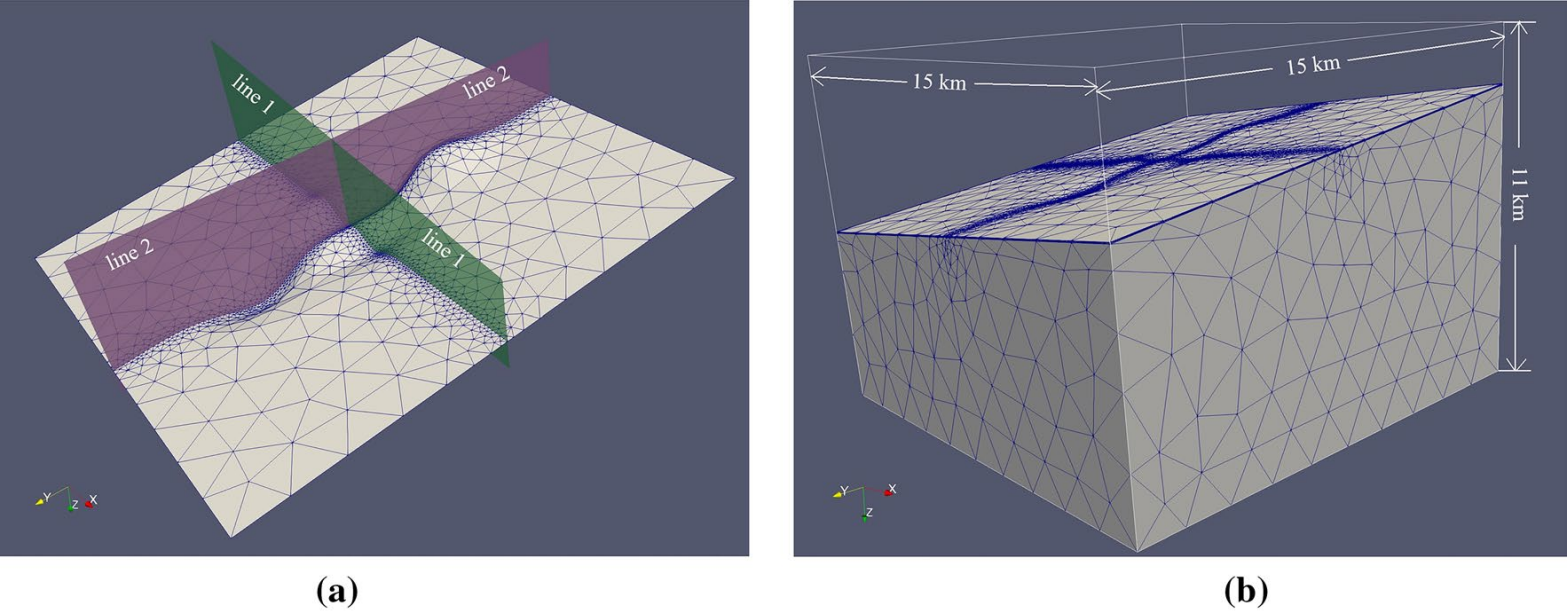
Three-dimensional geoelectric model with complex undulating terrain. (**a**) represents the undulating terrain and the two survey lines. (**b**) represents the whole three-dimensional model^40^.

In the high-frequency range, the hybrid mesh can obtain a high-precision EM response regardless of whether it is in flat terrain or undulating terrain with slopes. To further verify the adaptability of the hybrid mesh to undulating terrains, we design a three-dimensional geoelectric model with complex undulating terrain, which is referred to as the real terrain undulations (Fig. 15), and compare the accuracy of the MT response calculated by the hybrid mesh and the tetrahedral mesh. Here, the frequency is 1E4 Hz.

We set up three kinds of meshes, namely, a hybrid mesh, general tetrahedral mesh and dense tetrahedral mesh. The parameters of different meshes are shown in Table 5. The hybrid mesh and general tetrahedral mesh have the same horizontal element size, and their TriMESs are both 100 m. The horizontal element size of the dense tetrahedral mesh is smaller, and its TriMES is 20 m. The TriGRs in the three kinds of meshes are all 1.5. The thicknesses of the first layer in the three kinds of meshes are 15 m, 80 m and 20 m. To make the system matrix generated by the hybrid mesh and general tetrahedral mesh have similar DoF numbers, we set the growth rates of the hybrid mesh and general tetrahedral mesh to 1.5 and 1.34, respectively. The TetGR in the dense tetrahedral mesh is 1.5.

**Table 5:** Parameters of different meshes for the terrain relief model.

Mesh property	Horizontal size		Vertical size		NE	DoF	Time (min)	Memory (GB)
	TriMES (m)	TriGR	Thickness of the first layer (m)	PriGR/TetGR				
Hybrid mesh	100	1.5	15	1.0/1.5	53,108	500,815	6.617	44.4
General tetrahedral mesh	100	1.5	80	1.34	65,667	508,996	7.033	47.58
Dense tetrahedral mesh	25	1.5	20	1.5	135,669	1,046,150	16.833	97.45

**Figure 16:**
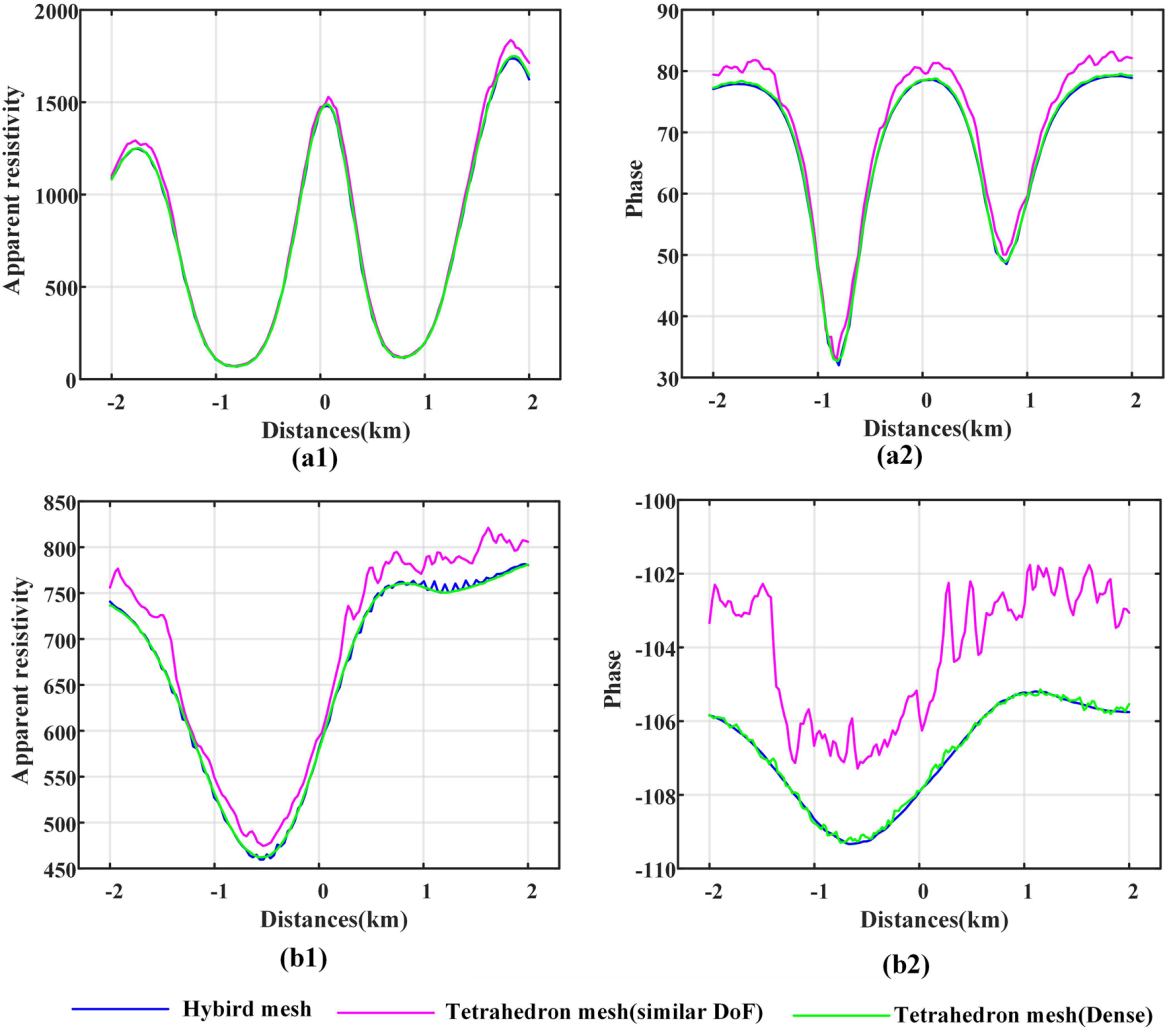
MT response calculation results of different meshes on survey line 1 to the terrain undulation model. (**a**) and (**b**) represent the MT response of the xy and yx components, respectively. (**a**1) and (**b**1) represent the apparent resistivity, and (**a**2) and (**b**2) represent the phase results.

**Figure 17:**
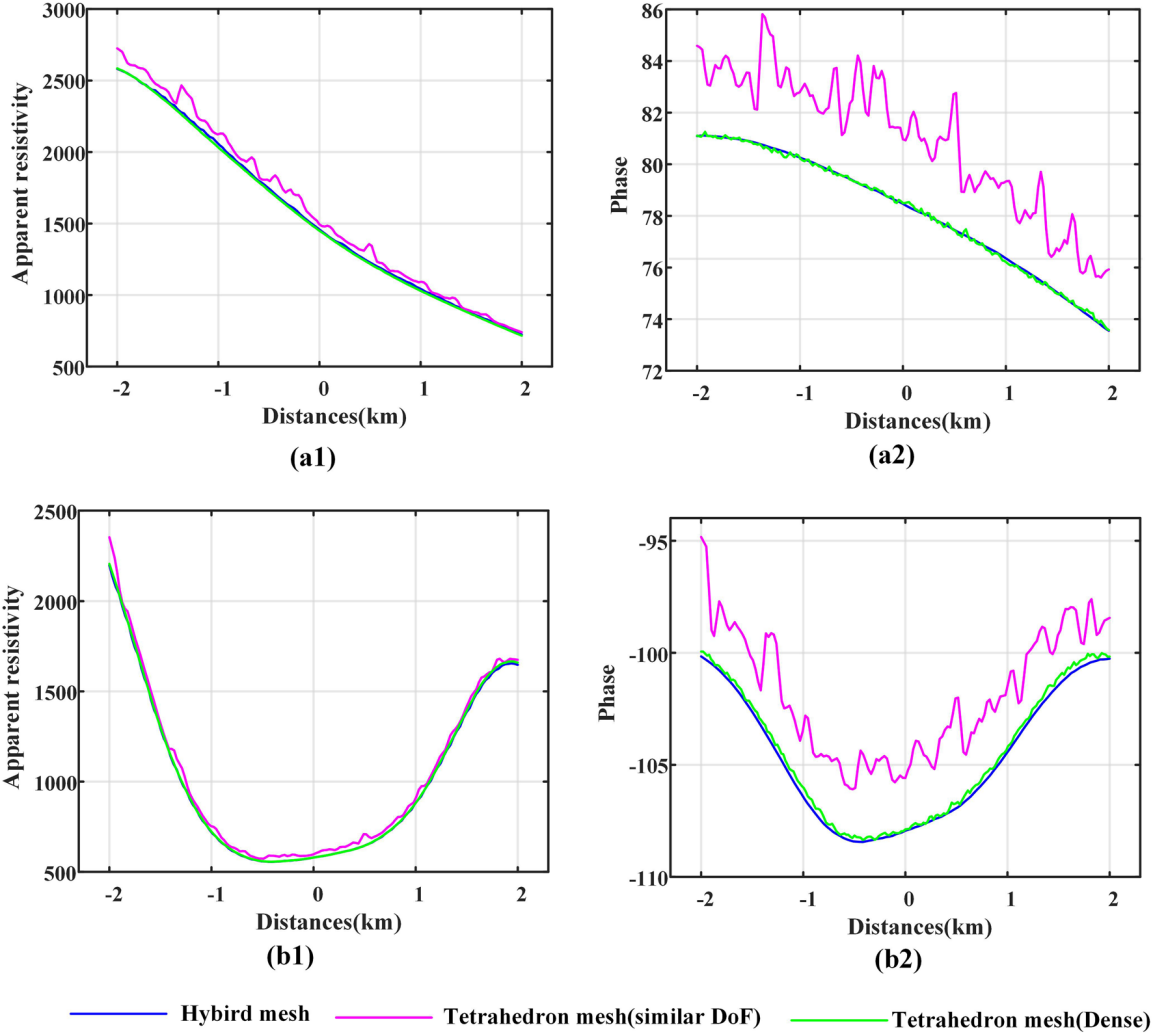
MT response calculation results of different meshes on survey line 2 to the terrain undulation model. (**a**) and (**b**) represent the MT response of the xy and yx components, respectively. (**a**1) and (**b**1) represent the apparent resistivity, and (**a**2) and (**b**2) represent the phase results.

The MT response results of the xy and yx components on the two survey lines are shown in Figs. 16 and 17. On survey line 1 (Fig. 16), the calculation result of the general tetrahedral mesh is the most inaccurate. The result of the hybrid mesh is closer to the result of the dense tetrahedral mesh. For the phase responses, the results of the dense tetrahedral mesh have obvious oscillations. This shows that the hybrid mesh can obtain stable calculation results while building a system matrix with a lower number of DoFs.

The response results on survey line 2 (Fig. 17) also show that when the DoF numbers are similar, the calculation results of the hybrid grid have higher accuracy than the calculation results of the general tetrahedral mesh. The dense tetrahedral grid can obtain more accurate calculation results, but the system matrix DoF number is nearly 2.6 times that of the hybrid grid. Additionally, compared with the phase results of the hybrid grid, the phase results of the dense tetrahedral grid also show stronger oscillations.

## Conclusions

We have proposed a hybrid mesh strategy for MT forward modeling with the FEM, which contains triangular prism elements and tetrahedral elements. The use of triangular prism elements to discretize the near-surface area can significantly reduce the amount of required NE, which is caused by mesh quality constraints for tetrahedrons. It can improve the computational efficiency of high-frequency data.

Different geoelectric models are used to analyze the accuracy and efficiency of the hybrid mesh in calculating the MT responses. The three-dimensional layered model and the DTM1 model verified their accuracy and applicability to general types of geoelectric models. A hybrid mesh can build an FE system matrix with fewer DoFs and obtain high accuracy in the solutions. Its superiority has also been verified in the example of terrain relief models.

The triangular prism elements have the flexibility of horizontal refinement, which is controlled by the triangular elements. It also has stability in terms of the element quality because the rectangular elements are not affected by the aspect ratio. We believe that application of this type of hybrid mesh is not limited to MT forward modeling but can also be useful to other geophysical electromagnetic calculation problems.

## Data Availability

The datasets used and/or analyzed during the current study are available from the corresponding author on reasonable request.

## Appendix A

### Shape functions of triangular prism element

The vector edge-shaped functions of the triangles are chosen as the Whitney-1 form basis function^46,47^ and can be represented as $\mathbf {W}$. The corresponding edge is determined by the node number indicated by the subscript $\mathbf {W}$. Two nodes at both ends determine the edge represented by $\mathbf {W}$. $\mathbf {W}$ can be expressed as:

33 $$\begin{aligned} \begin{array}{*{20}{c}} {{\mathbf{{W}}_{\mathrm{{23}}}}\mathrm{{ = }}{\xi _\mathrm{{2}}}\nabla {\xi _\mathrm{{3}}} - {\xi _\mathrm{{3}}}\nabla {\xi _\mathrm{{2}}}}\\ {{\mathbf{{W}}_{\mathrm{{31}}}}\mathrm{{ = }}{\xi _3}\nabla {\xi _1} - {\xi _1}\nabla {\xi _3}}\\ {{\mathbf{{W}}_{\mathrm{{12}}}}\mathrm{{ = }}{\xi _1}\nabla {\xi _2} - {\xi _2}\nabla {\xi _1}} \end{array} \end{aligned}$$

where ${{\xi _i}}$(i = 1, 2, 3) is a plane triangle nodal shape function established by Lagrangian interpolation. The Whitney-1 shape function represents the area coordinates of the triangle element. ${\nabla {\xi _i}}$(i = 1, 2, 3) represents the gradient vector of the triangular element. The calculation of these shape functions can directly refer to the element analysis of triangular elements in the two-dimensional case, but the three-dimensional shape function of the triangular prism cannot refer to the two-dimensional case. The local coordinate $\xi$(Fig. 2) in the height direction needs to be added to the shape function.

The vector edge-shaped functions for the bottom triangle edges (bottom vector functions) can be expressed as:

34 $$\begin{aligned} \begin{array}{*{20}{c}} {{\mathbf{{N}}_1}\mathrm{{ = }}\left( {1 - \zeta } \right) {\mathbf{{W}}_{\mathrm{{23}}}}}\\ {{\mathbf{{N}}_2}\mathrm{{ = }}\left( {1 - \zeta } \right) {\mathbf{{W}}_{\mathrm{{31}}}}}\\ {{\mathbf{{N}}_3}\mathrm{{ = }}\left( {1 - \zeta } \right) {\mathbf{{W}}_{\mathrm{{12}}}}} \end{array} \end{aligned}$$

The vector edge-shaped functions for the top triangle edges (top vector functions) can be expressed as:

35 $$\begin{aligned} \begin{array}{*{20}{c}} {{\mathbf{{N}}_4}\mathrm{{ = }}\zeta {\mathbf{{W}}_{\mathrm{{23}}}}}\\ {{\mathbf{{N}}_5}\mathrm{{ = }}\zeta {\mathbf{{W}}_{\mathrm{{31}}}}}\\ {{\mathbf{{N}}_6}\mathrm{{ = }}\zeta {\mathbf{{W}}_{\mathrm{{12}}}}} \end{array} \end{aligned}$$

The vector edge-shaped functions of the rectangle edges (volume vector functions) can be expressed as:

36 $$\begin{aligned} \begin{array}{*{20}{c}} {{\mathbf{{N}}_7}\mathrm{{ = }}{\xi _\mathrm{{2}}}\nabla \zeta }&{{\mathbf{{N}}_8}\mathrm{{ = }}{\xi _3}\nabla \zeta }&{{\mathbf{{N}}_9}\mathrm{{ = }}{\xi _1}\nabla \zeta } \end{array} \end{aligned}$$

The scalar node-based shape functions of the triangular prism element are:

37 $$\begin{aligned} {N_i} = \left\{ {\begin{array}{*{20}{l}} {\left( {1 - \zeta } \right) {\xi _i}}&{}{i = 1,2,3}\\ {\zeta {\xi _i}}&{}{i = 4,5,6} \end{array}} \right. \end{aligned}$$

### Shape functions of the pyramid prism element

The vector edge-shaped functions for the bottom rectangle edges can be expressed as:

38 $$\begin{aligned} \begin{array}{*{20}{c}} {\mathbf{{N}}_1^p = N_1^p\nabla \left( {N_2^p + N_3^p} \right) - N_2^p\nabla \left( {N_1^p + N_4^p} \right) }\\ {\mathbf{{N}}_2^p = N_2^p\nabla \left( {N_3^p + N_4^p} \right) - N_3^p\nabla \left( {N_2^p + N_1^p} \right) }\\ {\mathbf{{N}}_3^p = N_4^p\nabla \left( {N_2^p + N_3^p} \right) - N_3^p\nabla \left( {N_1^p + N_4^p} \right) }\\ {\mathbf{{N}}_4^p = N_1^p\nabla \left( {N_3^p + N_4^p} \right) - N_4^p\nabla \left( {N_1^p + N_2^p} \right) } \end{array} \end{aligned}$$

where ${\mathbf{{N}}^p}$represents the vector shape function of the pyramid element and ${N^p}$ represents the node shape function of the pyramid element. The expression of the vector shape function of the bevel of the pyramid element is^48,49^:

39 $$\begin{aligned} \begin{array}{*{20}{c}} \mathbf{{N}}_5^p = N_1^p\nabla N_5^p - N_5^p\nabla N_1^p\\ \mathbf{{N}}_6^p = N_2^p\nabla N_5^p - N_5^p\nabla N_2^p\\ \mathbf{{N}}_7^p = N_3^p\nabla N_5^p - N_5^p\nabla N_3^p\\ \mathbf{{N}}_8^p = N_4^p\nabla N_5^p - N_5^p\nabla N_4^p \end{array} \end{aligned}$$

The scalar node-based shape functions of the pyramid element are:

40 $$\begin{aligned} \begin{array}{*{20}{c}} {\begin{array}{*{20}{c}} {N_1^p = \frac{1}{4}\left[ {1 - \xi - \eta - \zeta + \frac{{\xi \eta }}{{1 - \zeta }}} \right] }&{}{N_2^p = \frac{1}{4}\left[ {1 + \xi - \eta - \zeta - \frac{{\xi \eta }}{{1 - \zeta }}} \right] } \end{array}}\\ {\begin{array}{*{20}{c}} {N_3^p = \frac{1}{4}\left[ {1 + \xi + \eta - \zeta + \frac{{\xi \eta }}{{1 - \zeta }}} \right] }&{}{N_4^p = \frac{1}{4}\left[ {1 - \xi + \eta - \zeta - \frac{{\xi \eta }}{{1 - \zeta }}} \right] } \end{array}}\\ {N_5^p = \zeta } \end{array} \end{aligned}$$
